# *Streptomyces* sp. VN1, a producer of diverse metabolites including non-natural furan-type anticancer compound

**DOI:** 10.1038/s41598-020-58623-1

**Published:** 2020-02-04

**Authors:** Hue Thi Nguyen, Anaya Raj Pokhrel, Chung Thanh Nguyen, Van Thuy Thi Pham, Dipesh Dhakal, Haet Nim Lim, Hye Jin Jung, Tae-Su Kim, Tokutaro Yamaguchi, Jae Kyung Sohng

**Affiliations:** 1 Department of Life Science and Biochemical Engineering, SunMoon University, 70 Sunmoon-ro 221, Tangjeong-myeon, Asan-si, Chungnam 31460 Republic of Korea; 2 Department of Pharmaceutical Engineering and Biotechnology, SunMoon University, 70 Sunmoon-ro 221, Tangjeong-myeon, Asan-si, Chungnam 31460 Republic of Korea

**Keywords:** Whole genome amplification, Mass spectrometry, Classification and taxonomy, Genome informatics, Bacteriology

## Abstract

*Streptomyces* sp. VN1 was isolated from the coastal region of Phu Yen Province (central Viet Nam). Morphological, physiological, and whole genome phylogenetic analyses suggested that strain *Streptomyces* sp. VN1 belonged to genus *Streptomyces*. Whole genome sequencing analysis showed its genome was 8,341,703 base pairs in length with GC content of 72.5%. Diverse metabolites, including cinnamamide, spirotetronate antibiotic lobophorin A, diketopiperazines cyclo-L-proline-L-tyrosine, and a unique furan-type compound were isolated from *Streptomyces* sp. VN1. Structures of these compounds were studied by HR-Q-TOF ESI/MS/MS and 2D NMR analyses. Bioassay-guided purification yielded a furan-type compound which exhibited *in vitro* anticancer activity against AGS, HCT116, A375M, U87MG, and A549 cell lines with IC_50_ values of 40.5, 123.7, 84.67, 50, and 58.64 µM, respectively. *In silico* genome analysis of the isolated *Streptomyces* sp. VN1 contained 34 gene clusters responsible for the biosynthesis of known and/or novel secondary metabolites, including different types of terpene, T1PKS, T2PKS, T3PKS, NRPS, and hybrid PKS-NRPS. Genome mining with HR-Q-TOF ESI/MS/MS analysis of the crude extract confirmed the biosynthesis of lobophorin analogs. This study indicates that *Streptomyces* sp. VN1 is a promising strain for biosynthesis of novel natural products.

## Introduction

Natural products (NPs) have been starting points of drug discovery for several decades. Major antimicrobials and chemotherapeutics entering clinical trials are often based on NPs^1,2^. Drugs with a natural origin can be produced as primary or secondary metabolites from versatile living organisms. Different microorganisms such as *Streptomyces*, myxobacteria and uncultured bacteria are major sources of such beneficial NPs^3,4^. Moreover, different metabolic engineering approaches and sophisticated techniques employing systems biology or synthetic biology can assist in harnessing the full potential of these bacteria in terms of productivity or creating diverse products^5,6^. Hence, there is renewed interest in mining microorganisms for new leads owing to the remarkable success of microbial metabolites as starting points for developing effective antibiotics, anticancer agents, and agrochemicals^7^.

*Streptomyces* are Gram-positive, aerobic bacteria in the order of *Actinomycetales* within the class of *Actinobacteria*. Genus *Streptomyces* was first proposed by Waksman and Henrici in 1943. It was classified into the family of *Streptomycetaceae* based on its morphology and cell wall chemotype^8^. Previous studies have shown that more than 74% of current antibiotics are derived from the genus *Streptomyces*^9^. Using integrated approaches of compound screening and drug development, *Streptomyces arsenal* has been found to be able to combat antibiotic resistance^5^. Multiple approaches such as ribosome engineering^10^ and genome mining^11^ have been used to find new secondary metabolites in old *Streptomyces* strains. Besides the effort to work on old strains to explore novel biosynthetic gene clusters (BGCs), isolating new bioactive strains from the environment is also a great way to find novel BGCs^12^. Recently, marine bacteria have been known as sources for many novel compounds. For example, lobophorins from marine bacteria are produced by *Streptomyces* strains^13,14^. Streptomycindole, a novel indole alkaloid compound with antibacterial activity, has been isolated from a marine *Streptomyces* sp. DA2 strain^15^.

**Figure 1:**
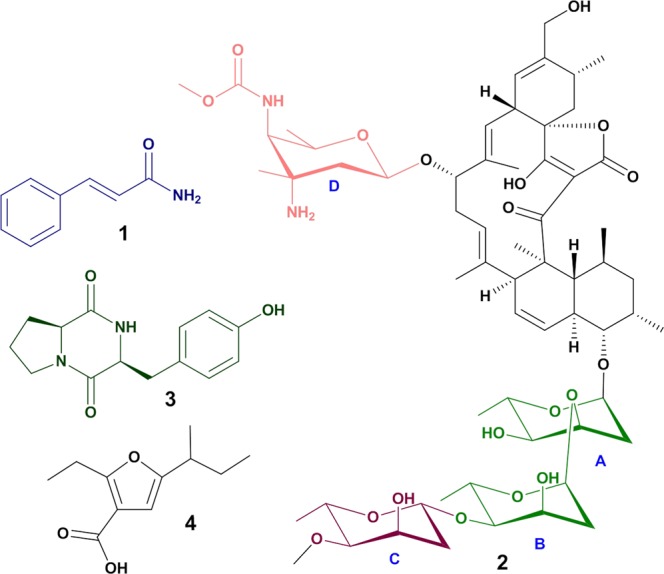
Structures of compounds **1**, **2**, **3**, and **4**. Letters “A-D” indicate four sugar units in lobophorin A.

In this study, we characterized a *Streptomyces* sp. VN1 isolated from the coastal region of Phu Yen Province, Da Nang, central Viet Nam. Based on phylogenetic, chemotaxonomic, and morphological characteristics, this strain belongs to genus *Streptomyces*. After a large-scale fermentation and bioassay-guided isolation, cinnamamide (**1**) lobophorin A (**2**) diketopiperazines cyclo-L-proline-L-tyrosine (**3**) and a furan-type compound (**4**) (Fig. 1) were characterized from fermentation broth of *Streptomyces* sp. VN1. Interestingly, the furan-type compound (**4**) exhibited anticancer activity against five types of tumor cell lines. Compound **4** effectively suppressed both migration and invasion of AGS cells. The ability of the strain to produce cinnamamide, antibacterial compounds **2** and **3**^16,17^, anticancer compound **4**, and diverse secondary metabolites make *Streptomyces* sp. VN1 an attractive target for bioactive compound screening.

## Materials and Methods

### Collection and isolation of strain

A sea sediment sample was collected offshore at a depth of 4.7 m at Da Nang Beach in Phu Yen Province, Viet Nam (latitude: 13°3′9.972″ N; longitude: 109° 11′57.444″ E). *Streptomyces* sp. VN1 was isolated by the direct spreading of sediment sample onto Gause’s synthetic agar plates^18^. After incubation at 28 °C for seven days, colonies were picked up and subcultured on modified Gause agar. The isolate strain was grown in modified Gause liquid medium containing yeast extract (3 g/L) supplemented as an additional nitrogen source. For long-term storage, pure colonies were transferred to starch-casein broth for seven days and cells were stored in 40% glycerol stock (v/v) at −80 °C. For compound production and characterization, the strain was grown on Marine broth-malt extract (MB cell).

### Morphological and phenotypic analyses

Morphological observation of spores and mycelia of *Streptomyces* sp. VN1 was done using an FE-SEM JEOL JSM-6700 (Jeol, Japan) after culturing on starch-casein agar media for ten days at 28 °C. Sole carbon sources at a concentration of 1% (w/v) and various salts at a concentration of 0.05% were tested for growth. Growth at various temperatures (4, 10, 15, 20, 24, 28, 30, 37, 42, 50, and 55 °C) and various pH conditions (pH 3–12; 1 pH unit intervals) was also evaluated in the same culture media.

### Chemotaxonomic analyses

Strain *Streptomyces* sp. VN1 was cultured aerobically on modified Gause liquid medium at 28 °C with shaking (160 rpm) for eight days. Cellular fatty acid compositions of the strain along with fatty acid methyl esters (FAMEs) were analyzed with a 6890 N Network GC system (Agilent Technology, USA) using the Sherlock Microbial Identification software package (version 6.1, database)^19^. Cell-wall amino acids and whole-cell carbohydrates were also extracted and analyzed as described previously^20,21^.

### Phylogenetic analysis

Strain *Streptomyces* sp. VN1 was cultivated in starch-casein broth. Genomic DNA extraction was carried out according to the standard procedure^22^. Cloned 16S rRNA was sequenced using the Sanger sequencing method. A 16S rRNA sequence of 1,467 nucleotides in length was obtained and deposited in GenBank (accession number: KU878019). This sequence was compared with sequences obtained from the online platform EzBioCloud (http://eztaxon-e.ezbiocloud.net/) through BLAST^23^. Phylogenetic analysis of the strain was performed to determine its taxonomic position within the genus of *Streptomyces*. For the analysis of genomic sequence, automated multi-locus species tree (autoMLST)^24^ pipeline was used to generate a phylogenetic based on alignment of default parameters (>100 core genes) present in closely related genomes using default parameters. Selected genomes were subjected to *in silico* DNA-DNA hybridization (DDH) and values were calculated using Genome-to-Genome Distance Calculator (GGDC)^25^ version 2.1 online.

### Genome sequencing, assembly, and annotation

Whole genome sequencing of *Streptomyces* sp. VN1 was performed using third-generation Pacific Biosciences (PacBio) RSII (Macrogen Corporation, Republic of Korea) sequencing system with single-molecule real-time (SMRT) analysis. HGAP3 was used to assemble PacBio long-reads. Filtered reads (8,558 reads, 966,726,581 bp) were then preassembled to generate long and highly accurate sequences (179,193 reads, 8,341,703 bp). For *de novo* assembly, Celera(R) Assembler was used to assemble sequences into a draft assembly. After that, the assembly was polished with a Quiver. The genome was annotated using Prokka (v1.12b). Transfer RNAs and ribosomal RNAs were analyzed using (tRNAs) tRNAscan-SE^26^ and RNAmmer^27^. Their functions were predicted according to databases of COG^28^ and EggNOG^29^. Secondary metabolite biosynthetic gene clusters were analyzed using antiSMASH 4.0^30^. The complete genome sequence of *Streptomyces* sp. VN1 was deposited in NCBI (accession number: CP036534).

### Mass spectrometric analysis of crude extraction sample

Marine broth-malt extract was used as fermentation medium. Briefly, 5 L of fermentation broth was extracted with 10 L of methylene chloride and evaporated under reduced pressure. The concentrate was collected using 50 mL of methanol to give residue A. The supernatant was extracted for the second time with 10 L ethyl acetate and its concentrate was collected using 50 mL MeOH to give residue B. These crude extracts were analyzed by ultra-high-performance liquid chromatography electrospray ionization quadrupole time of flight high-resolution mass spectrometry (UPLC-ESI-Q-TOF-HRMS) analysis using ACQUITY UPLC® (Waters Corporations, Milford, MA, USA) coupled with SYNAPT G2-S (Waters Corporations). UPLC®/HRMS analysis was performed on an ACQUITY UPLC® BEH C18 column (1.7 μm, 2.1 × 100 mm) at a flow rate of 0.3 mL min^−1^ using a gradient solvent mobile phase A (H_2_O, 0.1% trifluoroacetic acid) and B 0% to 100% ACN (0 to 12 min) at 35 °C. The injection volume was 10 µL. Conditions used for the high-resolution mass spectrometer equipped with an electrospray ionization source were: 3 kV of capillary voltage, 300 °C of desolvation gas temperature, and 600 L/h of desolvation gas flow rate. The mass range was set from *m/z* 50 to 1,700 in positive mode. MS/MS detection mode was set to a ramp trap collision high energy from 20 V to 40 V.

### Isolation of compounds from *Streptomyces* sp. VN1 and elucidation of their structures

Residues A and B were used as crude samples for further purification using a Dionex Ultimate 3000 UPLC (Thermo Fisher Scientific) equipped with a C_18_ column (YMC-Pack ODS-AQ, 150 × 20 mm^2^) connected to a UV detector (220, 254, 263, and 290 nm). Residue A was subjected to HPLC using a binary gradient of solvent A, 100% water; solvent B, 100% acetonitrile; 0% B to 100% B (linear gradient, 0–20 min), 100% B (20–25 min), 100% B to 0% B (25–30 min). The flow rate was 10 mL min^−1^ to give compound **1** (20.2 mg) and compound **2** (17.3 mg).

Residue B was subjected to HPLC using the following time program: solvent A, 100% water; solvent B, 100% acetonitrile; 0% B to 70% B (linear gradient, 0–20 min), 70% to 100% B (20–28 min), 100% B (28–30 min), 100% B to 0% B (30–40 min). The flow rate was 10 mL min^−1^ to give compound **3** (5.2 mg) and compound **4** (6.3 mg).

These purified compounds were dried, lyophilized, dissolved in methanol-*d*_4_, and subjected to 700 MHz using Bruker BioSpinnuclear magnetic resonance (NMR) (Billerica, USA) for analyses, including one-dimensional (1D) ^1^H-NMR,^13^C-NMR, and two-dimensional (2D) analyses.

IR (Infrared Spectroscopy) spectra were obtained using an EQUINOX 55 FT-IR spectrometer (BRUKER Optik GmbH, Wikingerstr. 13 76189 Karlsruhe, Germany). CD (Circular Dichroism) spectra were recorded using a JASCO J-715 spectrophotometer (Ochang, Republic of Korea). The CD spectrum was analyzed with a CDtoolX^31^ software.

### Cytotoxicity assay

Human cancer cell lines AGS, HCT116, A375SM, A549 and U87MG were obtained from the Korean Cell Line Bank (KCLB, Seoul, Korea). Human normal cell lines 267B1 and MRC-5 were purchased from the American Type Culture Collection (ATCC; Manassas, VA, USA). AGS gastric cancer and HCT116 colon cancer cells were maintained in RPMI 1640 medium containing 10% fetal bovine serum (FBS). A375SM melanoma and A549 lung cancer cells were grown in Dulbecco’s modified Eagle’s medium (DMEM) supplemented with 10% FBS. U87MG glioblastoma cells, normal cell lines of prostate epithelial (267B1) and lung fibroblast (MRC-5) were cultured in Minimum Essential Medium (MEM) containing 10% FBS. All cells were maintained at 37 °C in a humidified 5% CO_2_ incubator. For cell-growth assay, various cancer cells were plated into 96-well culture plates at density of 2 × 10^3^ cells/well. After compound **4** was added to each well at various concentrations (0, 0.78, 1.56, 3.12, 6.25, 12.5, 25, 50, 100, and 200 µM), cells were incubated at 37 °C for 72 h. Cell growth was measured using a 3-(4,5-dimethylthiazol-2-yl)-2,5-diphenyltetrazolium bromide (MTT) colorimetric assay. Briefly, 50 µL of MTT (2 mg/mL stock solution) was added and plates were incubated at 37 °C for an additional 4 h. After removal of medium, 100 µL of dimethyl sulfoxide (DMSO) was added to each well. The absorbance was measured at 540 nm using a microplate spectrophotometer (Thermo Scientific Multiskan® Spectrum). The cytotoxicity assays were performed on triplets and results are presented as mean values ± standard error (SE).

### Wound-healing assay

The migratory potential of AGS gastric cancer cells was analyzed using a wound-healing assay following published methodology^32^. Briefly, a confluent monolayer of AGS cells was scratched using a tip. Each well was then washed with PBS to remove nonadherent cells. Cells were then treated with compound **4** at 25 µM or 50 µM and then incubated at 37 °C for up to 24 h. The perimeter of the area with a central cell-free gap was confirmed under an optical microscope (Olympus). Dotted white line indicates the edge of the gap at 0 h. The wound-healing assays were performed on triplets and results are presented as mean values ± standard error (SE).

### Cell invasion assay

The experiment was performed following a previously described methodology^32^. Cell invasion was examined using Transwell chamber inserts with a pore size of 8.0 µm. Lower and upper sides of the polycarbonate filter were coated with gelatin (1 mg/mL) and Matrigel (3 mg/mL), respectively. AGS cells were seeded into the upper chamber of the filter while compound **4** at 25 µM or 50 µM was added to the lower chamber filled with medium. The chamber was incubated at 37 °C for 24 h. Cells were then fixed with methanol and stained with hematoxylin/eosin. The total number of cells that invaded the lower chamber of the filter was counted using an optical microscope (Olympus). The cell invasion assays were performed on triplets and results are presented as mean values ± standard error (SE).

### Statistical analysis

Results are presented as mean values ± standard error (SE). Student’s t-test was used to determine statistical significance between control and test groups. Statistical significance was considered at p < 0.05.

## Results

### Morphological and phenotypic characteristics

**Figure 2:**
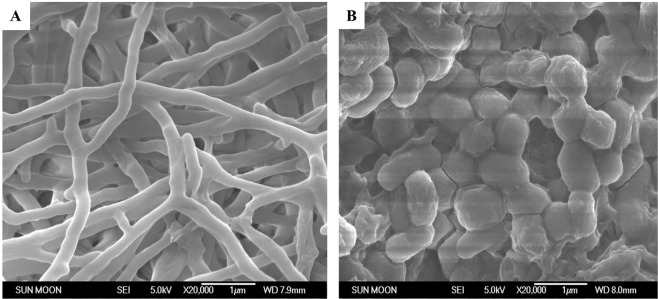
Scanning electron microscopic image showing the morphology of *Streptomyces* sp. VN1. (**A**) Rectiflexible mycelium; bar, 1 µm. (**B**) Smooth and oval-shaped spores; bar, 1 µm.

Scanning electron microscope observations revealed that the substrate mycelium of *Streptomyces* sp. VN1 was rectiflexible. When the culture of *Streptomyces* sp. VN1 reached maturity and adequate aerial mycelium was produced, aerial hyphae differentiated into short, straight to flexuous chains (Fig. 2A) with smooth surfaces. Its spores were found to be regular and smooth (Fig. 2B). *Streptomyces* sp. VN1 displayed survival on sole carbon sources of L-arabinose, fructose, mannitol, sucrose, xylose, lactose, and starch. Starch and yeast extract were preferred carbon source and nitrogen source, respectively (Table 1). *Streptomyces* sp. VN1 produced brownish to grayish mycelium with good sporulation on the media (i.e., Marine broth-malt extract medium) used for metabolite isolation. It showed growth at temperatures of 24 °C to 40 °C with pH between 4 and 9. Various salts had different effects on the growth of *Streptomyces* sp. VN1 (Supplementary Table S1).

**Table 1:** Morphological and physiological characteristics and carbon usage of *Streptomyces* sp. VN1.

Characteristics	*Streptomyces* sp. VN1
Spore morphology	Short, flexuous chains
Spore surface	Smooth
Production of diffusible pigments	−
Growth at 24 °C	+
Growth at pH 5	+
Growth at pH 11	−
**Growth on carbon sources**	
L-Arabinose	+
Fructose	+
Mannitol	+
Sucrose	+
Xylose	+
Lactose	+
Starch	+
**Growth on nitrogen sources**	
Cystine	+
L-Proline	+
Glycine	+
L-Asparagine	+

### Chemotaxonomic characteristics

Predominant fatty acids in *Streptomyces* sp. VN1 were C16:0 iso (23%), C15:0 antesio (19%), C15:0 iso (10%), and C16:0 (11%). Minor fatty acid methyl esters (FAME) such as C16:0 9-methyl (2.7%) and unsaturated fatty acids such as C16:1 iso (3.3%), C16:1 cis (7.5%), and C17:1 anteiso (2.1%) were detected (Supplementary Table S2). Fatty acids in genus *Streptomyces* are known to contain straight chains as well as iso- and anteiso-branched chains^20^. These FAMEs have been previously found in other species of *Streptomyces*^33^. Cell-wall peptidoglycans of *Streptomyces* sp. VN1 contained LL-diaminopimelic acid, typical of cell-wall type I. Galactose, arabinose, and xylose were detected as major carbohydrates in its whole-cell hydrolysate.

### Phylogenetic analysis and genome annotation

**Figure 3:**
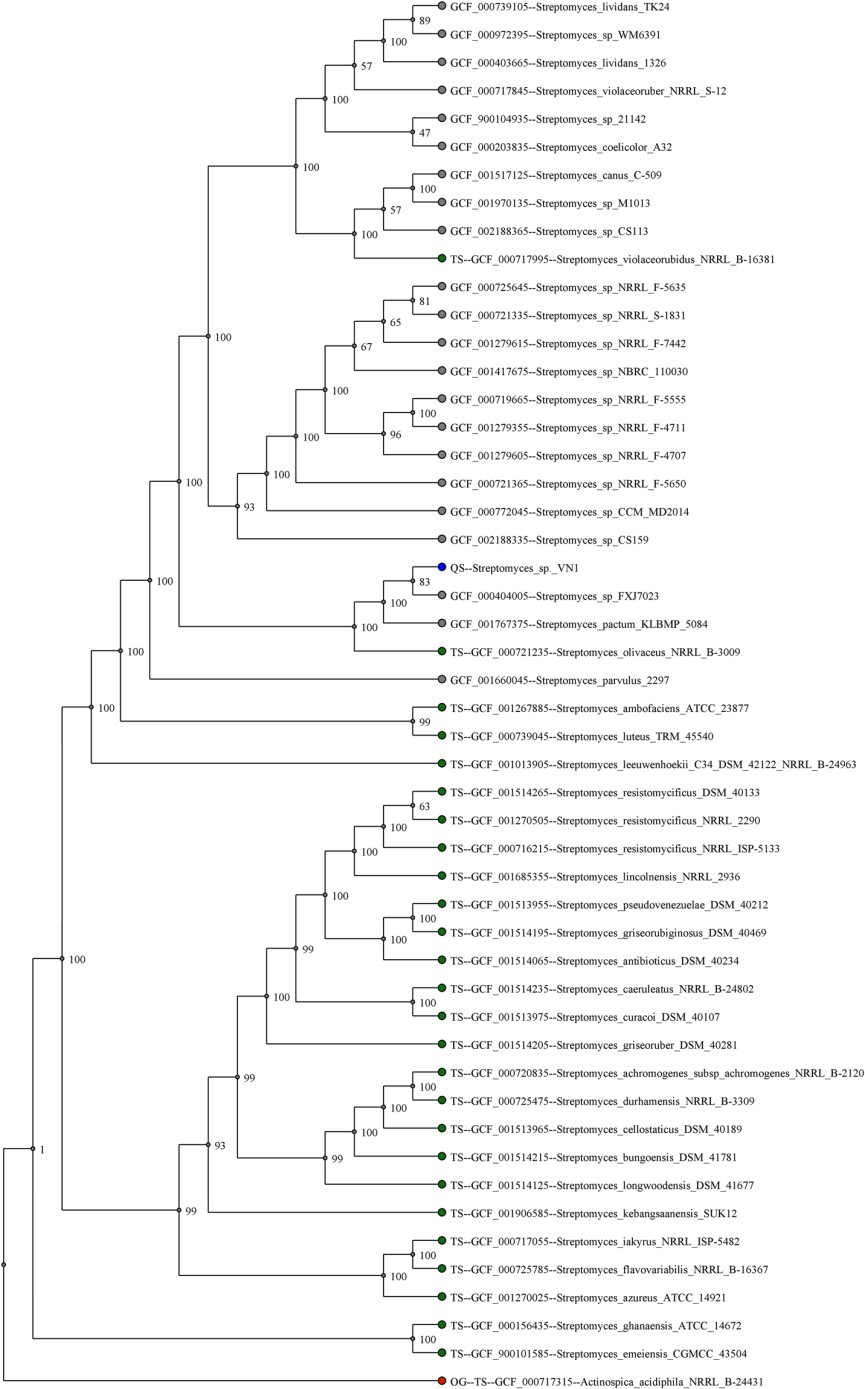
Molecular phylogenetic analysis of using default parameters (>100 core genes) by autoMLST. Bootstrap confidence levels are indicated at internodes whereas scale bar indicates nucleotide substitutions per nucleotide position.

Whole genome sequencing of *Streptomyces* sp. VN1 produced a total of 179,193 sequence reads, yielding a total consensus of 8,341,703 bp with GC content of 72.5% distributed within one main contig. There are 7,176 protein-coding genes, with an average ORF length of 1,018 bp. Coding density was about 87.57%. Within *Streptomyces* sp. VN1, 86 tRNAs and 18 rRNA operons were predicted (Table 2). Predicted proteins were annotated by blasting the eggNOG database. In eggNOG functional classification, 6,987 (97.36%) of 7,176 proteins were assigned. The following four top categories were classified: transcription, carbohydrate metabolism, amino-acid metabolism, and energy production (Supplementary Fig. S1). Genome analysis with autoMLST showed that this new isolate had the highest sequence similarities with *Streptomyces* sp. FXJ7.023 (GCF_000404005), *Streptomyces pactum* (GCF_001767375) and *Streptomyces olivaceus* (GCF_000721235) (Fig. 3). Estimated average nucleotide identities of *Streptomyces* sp. VN1 with genomes of *Streptomyces* sp. FXJ7.023, *Streptomyces pactum* and *Streptomyces olivaceus* were 99.4%, 99.2%, and 98.8%, respectively. Similarly, *in-silico* DDH values yielded high sequence similarities with *Streptomyces* sp. FXJ7.023 (99.87%), *Streptomyces pactum* (99.71%) and *Streptomyces olivaceus* (99.6%) (Supplementary Table S3). These data indicate that *Streptomyces* sp. VN1 is most closely related to *Streptomyces pactum* and *Streptomyces olivaceus*.

**Table 2:** General characteristics of the genome of isolated *Streptomyces* sp. VN1.

Sample	*Streptomyces* sp. VN1
Length (bp)	8,341,703
No. of reads	179,193
Coding density (%)	87.57
Average CDS length (bp)	1,018
No. of protein-coding genes	7,716
No. of tRNA genes	86
No. of rRNA	18
GC content	72.5

### *In silico* analysis of secondary metabolite biosynthesis pathways

Thirty-four secondary metabolite biosynthetic gene clusters were identified in the *Streptomyces* sp. VN1, including type I polyketide synthases (T1PKS), type II polyketide synthases (T2PKS), type III polyketide synthases (T3PKS), non-ribosomal peptide synthetases (NRPS), terpenes, lassopeptides, thiopeptides, lanthipeptides, indoles, siderophores, bacteriocins, T3PKS-NRPS, T1PKS-NRPS, and other products (Supplementary Table S4). BGCs for common secondary metabolites found in *Streptomyces* including 2-methylisoborneol, ectoine, melanin, hopene, and coelichelin were found to be present in *Streptomyces* sp. VN1. Analysis results showed that *Streptomyces* sp. VN1 contained seven of PKS, terpene, and NRPS gene clusters related to antibiotics, displaying a similarity of more than 40%, including lobophorin A, carotenoid, friulimicin, xiamycin, enterocin, divergolide, and informatipeptin biosynthetic gene clusters (Table 3).

**Table 3:** Overview of *Streptomyces* sp. VN1 genome analysis by antiSMASH of 17 secondary metabolites of biosynthetic gene clusters and seven PKS and NRPS gene clusters sharing similarity of more than 40%.

No	Cluster	Type	From	To	Most similar known biosynthetic gene cluster (percent of similarity)	Reference strain	Accession number
**1**	1	Oligosaccharide-T1PKS-T3PKS-NRPS	10491	225803	Lobophorin A (96%)	*Streptomyces* sp. FXJ7.023	JX306680
**2**	2	Terpene	237497	258555	2-Methylisoborneol (100%)	*Streptomyces griseus*	AP009493
**3**	5	Terpene	539486	563714	Carotenoid (54%)	*Streptomyces avermitilis*	AB070934
**4**	9	Ectoine	1684875	1695273	Ectoine (100%)	*Streptomyces anulatus*	AY524544
**5**	10	Melanin	2650272	2660898	Melanin (100%)	*Streptomyces coelicolor* A3(2)	AL645882
**6**	11	Lassopeptide	2720839	2743360	SSV-2083 (50%)	*Streptomyces sviceus*	NZ_CM000951
**7**	12	Siderophore	2756614	2768407	Desferrioxamine_B (83%)	*Streptomyces coelicolor A3(2)*	AL645882
**8**	15	Lantipeptide	4240796	4272172	SBI-06990 alpha/SBI-06989 beta (50%)	*Streptomyces bingchenggensis*	CP002047
**9**	17	Terpene	5321623	5342708	Albaflavenone B (100%)	*Streptomyces coelicolor* A3(2)	AL645882
**10**	18	T2PKS	5408372	5450926	Spore_pigment (66%)	*Streptomyces avermitilis*	AB070937
**11**	20	T1PKS-NRPS	6048796	6212376	Friulimicin (75%)	*Actinoplanes friuliensis*	AJ488769
**12**	21	T1PKS-NRPS	6260144	6309491	Xiamycin (77%)	*Streptomyces* sp. SCSIO 02999	JQ812811
**13**	26	T2PKS	6650019	6692435	Enterocin (95%)	*Streptomyces maritimus*	AF254925
**14**	28	Terpene-NRPS	7137719	7221298	Hopene (92%)	*Streptomyces coelicolor* A3(2)	AL645882
**15**	29	T1PKS	7275849	7358369	Divergolide (100%)	*Streptomyces* sp. HKI0576	HF563079
**16**	30	Bacteriocin	7687326	7697541	Informatipeptin (42%)	*Streptomyces viridochromogenes* DSM 40736	GG657757
**17**	32	NRPS	7898917	7949836	Coelichelin (100%)	*Streptomyces coelicolor* A3(2)	AL645882

The number of BGCs is determined with antiSMASH and ClusterFinder OFF.

**Figure 4:**
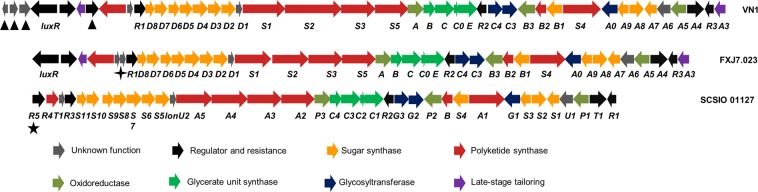
Genetic organization of the lobophorin biosynthetic gene cluster of *Streptomyces* sp. VN1 (GenBank: SUB5241063), lobophorin gene cluster of *Streptomyces olivaceus* FXJ7.023 (GenBank: JX306680), and lobophorin gene cluster of *Streptomyces* sp. SCSIO 01127 (GenBank: KC013978). “▲”; “”; “★” denote unique genes in *Streptomyces* sp. VN1, *Streptomyces olivaceus* FXJ7.023, and *Streptomyces* sp. SCSIO 01127, respectively.

Cluster 1 contained a oligosaccharide-T1PKS-T3PKS-NRPS that was highly similar to lobophorin BGCs from *Streptomyces* sp. FXJ7.023 and *Streptomyces* sp. SCSIO 01127 (Table 3). A detailed analysis of the lobophorin A BGC of *Streptomyces* sp. VN1 indicated the involvement of 43 ORFs (open reading frame) and five catalytic domains related to polyketide synthases (Fig. 4). Lobophorin A biosynthesis gene clusters of *Streptomyces* sp. VN1 is highly similar to previously characterized BGCs which shared around 87–100% identities with corresponding genes in *Streptomyces* sp. FXJ7.023 and *Streptomyces* sp. SCSIO 01127 (Supplementary Table S5A). Next to the lobophorin A biosynthetic gene cluster was predicted for the T3PKS-NRPS biosynthetic gene cluster. It contained 48 ORFs, one catalytic domain related to chalcone/stilbene synthase, two catalytic domains related to polyketide synthases and three catalytic domains related to non-ribosomal peptide synthases. Recently, this T3PKS-NRPS biosynthetic gene cluster has been characterized as being responsible for synthesizing totopotensamide^34^. In-depth bioinformatic analysis of the putative biosynthesis gene cluster for totopotensamide in *Streptomyces* sp. VN1 is highly similar to previously characterized BGC which showed about 96–100% similarity with corresponding genes in *Streptomyces* sp. SCSIO 02999 (Supplementary Table S5B). It has been proposed that *orf51* gene in *Streptomyces* sp. VN1, *lobR3* in *Streptomyces* sp. FXJ7.023, and *lobR1* in *Streptomyces* sp. SCSIO 01127 regulate lobophorin biosynthesis^35,36^. However, *lobR1* has been recently annotated as *totR5* belonging to the *tetR* family transcriptional regulator that is responsible for regulating totopotensamides synthesis^37^ in *Streptomyces* sp. SCSIO 02999. Besides, the end of cluster 1 in *Streptomyces* sp. VN1 contained four ORFs annotated for four regulator genes and a unique ORF (*orf98*) annotated for N-acetyltransferase (Supplementary Table S5B).

Analysis results suggested that cluster five is highly similar with previously characterized carotenoid biosynthesis gene cluster. It shared 54% similarity with an existing cluster in *Streptomyces avermitilis* (Supplementary Fig. S2). This cluster contained 20 ORFs for biosynthesis of carotenoid in *Streptomyces* sp. VN1. Core ORFs biosynthesis such as phytoene synthase, lycopene cyclase, dehydrogenase, and methyltransferase were annotated. This cluster of *Streptomyces* sp. VN1 was predicted to have unique ORFs annotated as triacylglycerol lipases and short chain dehydrogenase/reductases (SDR), indicating that *Streptomyces* sp. VN1 might have the potential to synthesize carotenoid analogs.

Furthermore, two NRPS-hybrid synthases including a T1PKS-NRPS gene cluster showed high similarities to friulimicin and xiamycin biosynthetic gene cluster present in *Actinoplanes friuliensis* and *Streptomyces* sp. SCSIO 02999. From the friulimicin biosynthetic gene cluster, 12 core ORFs were annotated. The putative friulimicin biosynthetic gene cluster from *Streptomyces* sp. VN1 contained an additional condensation domain containing protein, a phosphopantetheine-binding domain containing protein synthetase, and an AMP-dependent protein and ligase (Supplementary Fig. S3). Therefore, *Streptomyces* sp. VN1 has high potential to produce friulimicin analogs. The putative xiamycin biosynthesis gene cluster from *Streptomyces* sp. VN1 was found to contain 33 ORFs (Supplementary Fig. S4). In this gene cluster, core and additional biosynthetic genes were annotated to polyprenyl synthetase, cytochrome P450, acyl-CoA dehydrogenase, and a crotonyl-CoA reductase/alcohol dehydrogenase including additional dehydrogenase. Compared to the most-similar known cluster of *Streptomyces* sp. SCSIO 02999, this gene cluster of *Streptomyces* sp. VN1 was found to contain a unique ORF annotated as hybrid polyketide synthetase and non-ribosomal peptide synthase. This indicates that the strain *Streptomyces* sp. VN1 might have potential to synthesize xiamycin analogs.

In bioinformatics analysis of the *Streptomyces* sp. VN1 genome, one T2PKS was annotated for an enterocin biosynthesis gene cluster that was 95% similar to the enterocin biosynthesis gene cluster from *Streptomyces maritimus* (Supplementary Fig. S5). In this putative enterocin biosynthesis gene cluster, eight core ORFs that contained an additional methyltransferase were annotated. Thus, *Streptomyces* sp. VN1 might produce enterocin derivatives.

Additionally, *Streptomyces* sp. VN1 was predicted to harbor T1PKS pathways. The T1PKS gene cluster is related to biosynthesis of divergolide. It showed 100% homology with the existing cluster of *Streptomyces* sp. HKI0576 (Supplementary Fig. S6).

Interestingly, biosynthetic gene cluster thirty-two was predicted to be related to bacterocin informatipeptin biosynthesis. The putative informatipeptin biosynthesis cluster of *Streptomyces* sp. VN1 displayed 42% homology with an existing cluster from *Streptomyces viridochromogenes* DSM 40736 (Supplementary Fig. S7). In the genome of *Streptomyces* sp. VN1, only three ORFs (type A lantipeptide, protease, and PAS/PAC sensor protein) were found to be related to informatipeptin biosynthesis.

Furthermore, the T3PKS gene cluster was predicted to be related to herboxidiene biosynthesis (Supplementary Fig. S8). A comparison of the putative herboxidiene biosynthetic gene cluster with *Streptomyces chromofuscus* A7847 indicated that numerous genes in this cluster could not be annotated. This gene cluster only shared 2% homologies with the herboxidiene biosynthetic gene cluster from *Streptomyces chromofuscus* A7847.

The putative NRPS-nucleoside biosynthetic gene cluster from *Streptomyces* sp. VN1 was found to contain 34 ORFs related to nogalamycin biosynthesis (Supplementary Fig. S9). A comparison of the nogalamycin biosynthetic gene cluster from *Streptomyces nogalater* indicated that numerous genes in this cluster could not be annotated. This gene cluster only shared 40% homology with the nogalamycin biosynthetic gene cluster from *Streptomyces nogalater*.

### Structural elucidation of major compounds

UV spectra and the HR-MS data of compounds **1** to **3** were as follows. Compound **1** (in CH_3_CN: H_2_O: trifluoroacetic acid) revealed absorption maxima at 200.73 and 273.73 nm with HR-MS *m/z* 148.0755 (calculated for C_9_H_9_NO, [M + H]^+^ 148.0762) (Supplementary Fig. S10A). The UV spectrum of compound **2** (in CH_3_CN: H_2_O: trifluoroacetic acid) revealed absorption maxima at 210.73 and 262.73 nm with HR-MS *m/z* 1,157.6379 (calculated for C_61_H_92_N_2_O_19_, [M + H]^+^ 1,157.6367) (Supplementary Fig. S10B). The UV spectrum of compound **3** (in CH_3_CN: H_2_O: trifluoroacetic acid) revealed absorption maxima at 201.73 and 266.73 nm with HR-MS *m/z* 261.1249 (calculated for C_14_H_16_N_2_O_3_, [M + H]^+^ 261.1243) (Supplementary Fig. S10C). Compounds **1**, **2**, and **3** were found to be cinnamamide, lobophorin A, and cyclo-L-proline-L-tyrosine, respectively, by comparing ^1^H (700 MHz, methanol-*d*_4_) and ^13^C (176 MHz, methanol-*d*_4_) spectra data (Supplementary Table S6) with data reported in the literature^13,17,37^.

**Figure 5:**
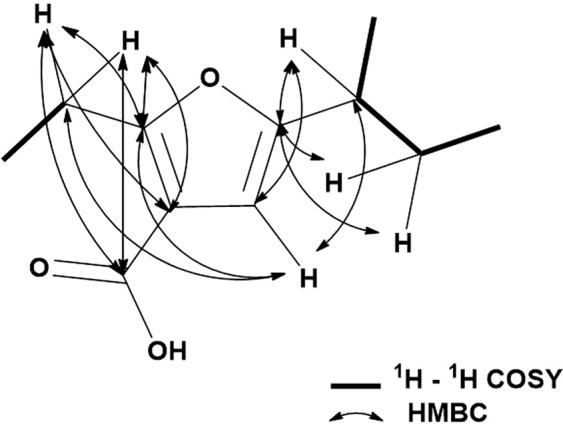
Selected key ^1^H -^1^H COSY (bold line) and HMBC (H  C) correlations of furan type-compound **4**.

According to spectrophotometric analysis, the UV spectrum of compound **4** (in CH_3_CN: H_2_O: trifluoroacetic acid) revealed absorption maxima at 201.73 and 290.73 nm with HR-MS *m/z* 197.1271 (calculated for C_11_H_16_O_3_, [M + H]^+^ 197.1172) (Supplementary Fig. S11). The IR spectrum of **4** revealed a conjugated carboxylic acid-carbonyl stretching-vibration absorption at 1,664 cm^−1^, a CH_3_- asymmetric stretching-vibration absorption at 1,966 cm^−1^, a CH_3_- symmetric stretching-vibration absorption at 2,874 cm^−1^, a CH_2_-asymmetric stretching-vibration absorption at 2,930 cm^−1^, and a carboxylic acid hydrogen bonding broad −OH stretching-vibration absorption at 3,400–2,400 cm^−1^ (Supplementary Fig. S12). ^1^H (700 MHz, methanol-*d*_4_) and ^13^C (176 MHz, methanol-*d*_4_) NMR (Table 4) led to structural conclusions for compound **4**. The overall structure of **4** was established mainly based on ^1^H-^1^H correlation spectroscopy (COSY) and heteronuclear multiple-bond correlation (HMBC) correlations (Fig. 5). In the ^1^H-^1^H COSY spectrum, cross-peaks between δ_H_ 1.22 ppm and 2.48 ppm, δ_H_ 2.48 ppm and 1.57 ppm, 2.48 ppm and 1.70 ppm, 1.57 ppm and 0.91 ppm, 1.70 ppm and 0.91 ppm indicated three C-C bonds from C-9 to C-12. The correlation peak of between δ_H_ 2.42 and 1.05 indicated C-C bond of C-6 and C-7 (Supplementary Fig. S13C). In the HMBC spectrum, observed correlated signals between δ_H_ 5.99 (s) and three carbon (δ_C_ 103.67, 166.88 and 167.15) indicated the skeleton of C-2, C-3, C-4, and C5 based on intensities of cross-peaks. In addition, the connection of C-5 and C-10 was confirmed based on the finding of cross-peaks showing that H-9, H-10, H-11′, and H-11″ were correlated to C-5 by C-H long-range coupling. Moreover, the connection of C-2 and C-6 was confirmed from the finding of cross-peaks showing that H-6 was correlated to C-2 by C-H long-range coupling. The cross-peaks of H-6 and δ_C_ 167.48 (C-8) indicated the bonding of C-3 and C-8 as the carbonyl carbon (Supplementary Fig. S13F). ^13^C NMR chemical shift values of C-5 and C-2 were around 167 ppm as the region of the carbonyl carbon of ester, amide, and carboxylic acid group. If two carbons C-5 and C-2 build the ketone form, ^13^C chemical shifts should appear at around 200 ppm. However, the ^13^C spectrum did not show chemical shifts at around 200 ppm. This observation suggests that C-5 and C-2 are carbons of the double bond with oxygen attached. The number of this oxygen should be one based on HR-MS data. This observation led to the conclusion that it has a shape of five-membered ring formations of furan. Thus, the structure of **4** was completely elucidated to be 5-(*sec*-butyl)−2-ethylfuran-3-carboxylic acid. Absolute configurations experimental CD spectra of compound **4** showed cotton effect at wavelength of 200, 205, 225, and 290 nm. The positive cotton effect in π−π* transition of –C=C- was strong at 205 nm Δε + 15.12 (mdeg). That of –C=C–C=C– was slightly broad at 290 nm Δε + 1.56 (mdeg). The negative cotton effect negative in the π−π* transition of the -OH was strong at 200 nm Δε −19.4 (mdeg). In the n−π* transition of the –COOH, it was at 225 Δε −6.7 (mdeg) (Supplementary Fig. S14).

**Table 4:** ^1^H- and ^13^C-NMR data of compound **4** in methanol-*d*_4_.

No	^13^C (ppm)	^1^H (ppm)	Intensities
1			
2	167.15		
3	103.67		
4	99.51	5.99 (s)	1 H
5	166.88		
6	15.89	2.42 (q, *J* = 7.4)	2 H
7	11.50	1.05 (t, *J* = 7.4)	3 H
8	167.48		
9	16.89	1.22 (d, *J* = 6.9)	3 H
10	39.55	2.48 (tq, *J* = 6.9, 7.0)	1 H
11	27.10	1.57 (m) 1.70 (m)	1 H 1 H
12	10.51	0.91 (t, *J* = 7.5)	3 H

### Identification of lobophorin A analogs by mass spectrometry

**Figure 6:**
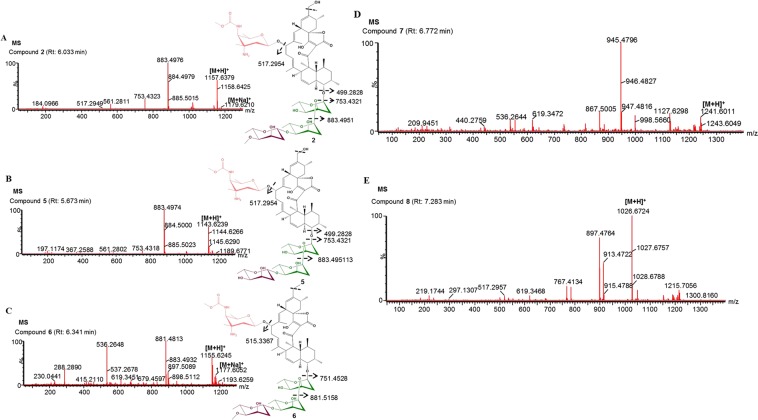
HR**-**MS and MS/MS analyses of lobophorin analogs. (**A**) The component of lobophorin A generates a [M + H]^+^ ion at *m/z* 1,157.6379. (**B**) The component of compound **5** (demethylation of lobophorin A) generates a [M + H]^+^ ion at *m/z* 1,143.6239. (**C**) The component of compound **6** (dehydroxylation of lobophorin A) generates a [M + H]^+^ ion at *m*/*z* 1,155.6245. (**D**) The component of compound **7** generates a [M + H]^+^ ion at *m/z* 1,2041.6011. (**E**) The component of compound **8** generates a [M + H]^+^ ion at *m/z* 1,026.6724.

We further analyzed mass spectra of crude extract to determine whether predicted secondary metabolites could be detected. Mass spectra data were obtained in positive mode. From HR-MS/MS profiles, metabolic substances in crude extract samples were found to be correlated with the mass-to-charge ratio (*m/z*) of molecular ions. Mass profiles of secondary metabolites existing in isolated strains were compared with genome mining data. Herein, we identified lobophorin A and its analogs in the crude extract through this technique. The proposed fragment of lobophorin A was analyzed based on the fragmentation of kijanimicin^38^ (Supplementary Fig. S15A). In the mass profile of lobophorin A presented in Fig. 6A, fragments corresponded to [M + H]^+^/[M + Na]^+^ 1,157.6379/1,179.6210, (M-B-C + H)^+^ 883.4976, (M-A-B-C + H)^+^ 753.4324, and (553-2H_2_O)^+^ 517.2949, in full accord with the structure. Detailed fragmentation of MS/MS of lobophorin A is shown in Supplementary Fig. S15B. Compound **2** (lobophorin A, Rt: 6.033 min) and lobophorin A analogs namely compound **5** (demethylated of lobophorin A, retention time, Rt: 5.673 min), compound **6** (dehydroxylated lobophorin A, Rt: 6.341 min), compound **7** (Rt: 6.772 min) and compound **8** (Rt: 7.283 min), were detected (Fig. 6) in fraction A. Possible structures and fragmentation of compounds **5** and **6** were compared with the mass profile of lobophorin A and previously described structures^36,39^. These compounds displayed UV spectrum and fragmentation similar to those of lobophorin A (Supplementary Fig. S15). The first analog, compound **5** showed [M + H]^+^ ions at *m/z* 1,143.6239 (calculated for C_60_H_91_N_2_O_19_:[M + H]^+^ 1,143.6211) (Fig. 6B). This analog has been verified and characterized by MS/MS and NMR in a previous study^39^. Peaks generated by MS/MS analysis showed that main fragment ions were at 883.4966, 753.4326, and 517.2950 (Supplementary Fig. S15C). The second analog, compound **6** showed [M + H]^+^/[M + Na]^+^ ions at *m/z* 1,155.6245/1,177.6052 (calculated for C_62_H_95_N_2_O_18_:[M + H]^+^ 1,155.6574; C_62_H_94_N_2_O_18_Na: [M + Na]^+^ 1,177.6399) (Fig. 6C). Peaks generated by MS/MS analysis showed that main fragment ions were at 881.4817, 751.4147, 619.3461, and 515.2808 (Supplementary Fig. S15D). Interestingly, the third derivative, compound **7** showed [M + H]^+^ ions at *m/z* 1,2041.6011 (Fig. 6D). Peaks generated by MS/MS analysis showed that main fragment ions were at 945.4792, 867.5002, 737.5001, 619.3464, 517.2946, and 499.2837 (Supplementary Fig. S15E). It was noteworthy that the fourth analog, compound **8** showed [M + H]^+^ ions at *m/z* 1,026.6724 (Fig. 6E). Peaks generated by MS/MS analysis showed that main fragment ions were at 895.5777, 783.4073, 619.3467, 517.2947, and 455.3103 (Supplementary Fig. S15F). To the best of our knowledge, there is no structural reference for compound **7** or **8**. They might be new analogs of lobophorin. We also analyzed mass spectra of crude extract residue B. However, we could not correlate the data with the mass of any secondary metabolite (data not shown).

### Anticancer activity

**Figure 7:**
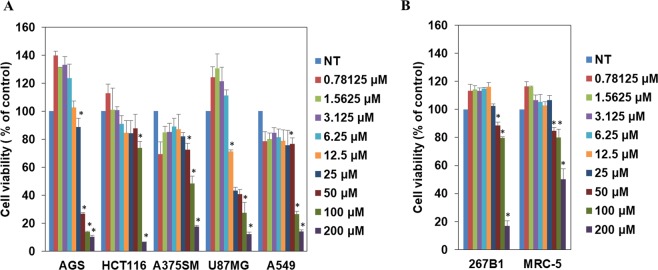
*In vitro* growth inhibitory activities of compound **4** against different cell lines. (**A**) cancer cell lines, (**B**) normal cell lines. NT: no treatment. *p < 0.05 vs. control.

**Figure 8:**
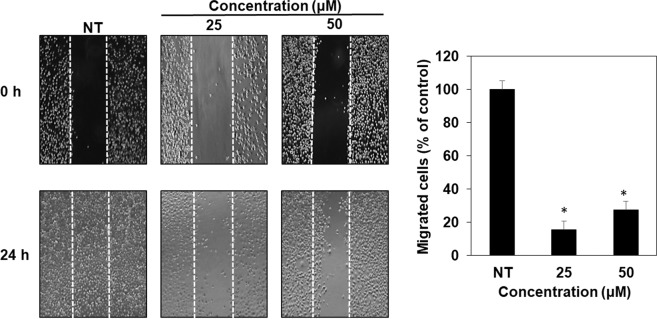
Cell migration inhibitory activity of furan-type compound against AGS cancer cells. NT: no treatment. *p < 0.05 vs. control.

**Figure 9:**
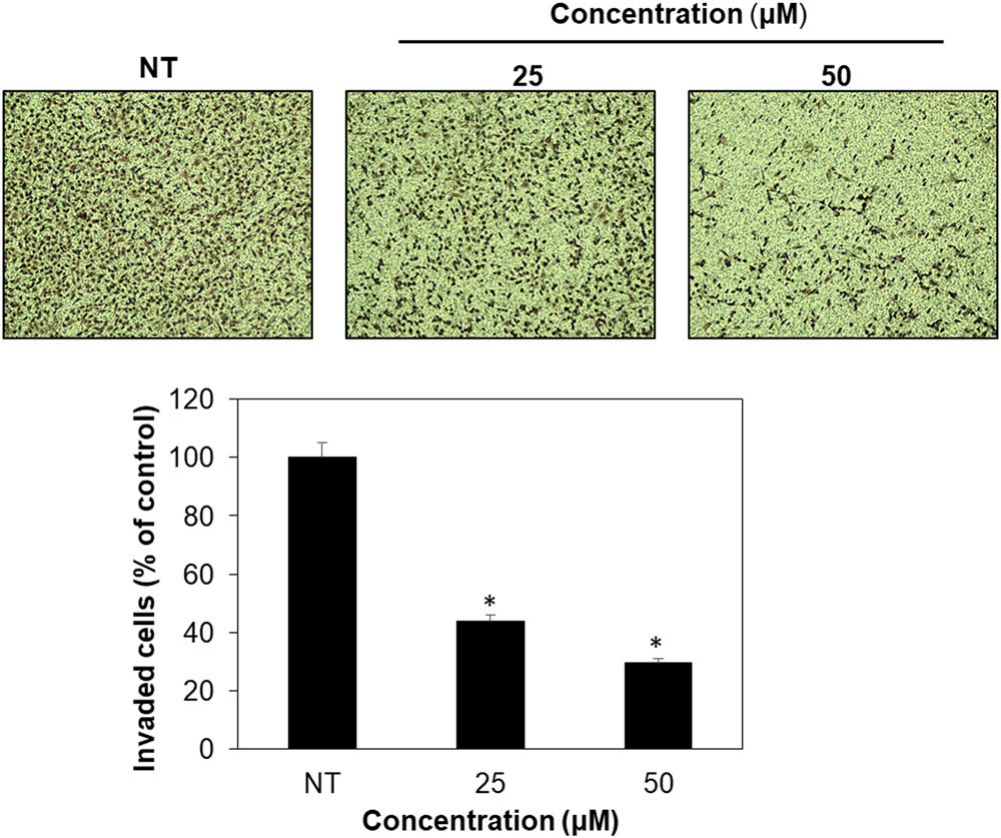
Cell invasion inhibitory activity of furan-type compound against AGS cancer cells. NT: no treatment. *p < 0.05 vs. control.

To assess whether compound **4** might have anticancer activity, we evaluated the inhibitory effect of compound **4** on the growth of different tumor cell lines. Compound **4** showed anti-proliferative activities against five types of cancer cell lines (Fig. 7A). This compound exhibited more sensitive growth-inhibitory activities for gastric adenocarcinoma (AGS), glioblastoma (U87MG), and lung cancer (A549) than for melanoma (A375SM) and colon cancer cells (HCT116) (Table 5; Fig. 7A). Compound **4** more effectively inhibited the growth of cancer cell lines than that of normal cell lines (267B1 and MRC-5) at tested doses (Table 5; Fig. 7B). Besides its inhibitory effect against cancer cell growth, the anti-metastatic activity of compound **4** against AGS gastric cancer cell line was also determined through migration and invasion assays. We found that compound **4** effectively suppressed both the migration and invasion of AGS cells after 24 h of treatment at concentrations of 25 and 50 µM (Figs. 8 and 9). These data suggest that compound **4** possesses an anticancer activity by inhibiting the growth and metastasis abilities of cancer cells.

**Table 5:** IC_50_ value of compound **4**.

Compound 4	AGS (Gastric cancer)	HCT116 (Colon cancer)	A375SM (Melanoma)	U87MG (Glioblastoma)	A549 (Lung cancer)	267B1 (prostate epithelial)	MRC-5 (lung fibroblast)
IC_50_ (µM)	40.5	123.7	84.67	50	58.64	157.2	>200

## Discussion

Marine *Streptomyces* are valuable sources of various secondary metabolites for drug discovery. In this study, we found that *Streptomyces* sp. VN1 as a new strain produced many active compounds, including cinamamide, lobophorin A, cyclo-L-proline-L-tyrosine, and a furan-type compound based on HR-MS/MS and 2D-NMR studies. Besides these compounds, possible analogs of lobophorin A were found based on HR-MS/MS analysis. We found that fragments of those compounds were similar to a fragment of lobophorin A. However, in mass profiles they appeared in trace amounts. Thus, we were unable to elucidate structures of these compounds.

Cinamamide is the product of phenylalanine ammonia lyase. It has been examined in the biosynthesis of cinnamamide in cultures of *Streptomyces verticillatus* using L-phenylalanine-*carboxyl*-[^14^C]^40^. Cinamamide is a nonlethal chemical repellent^41^. Recently, cinamamide derivatives have been synthesized. They show potential activities in both peripheral and central nervous systems, including antiepileptic, antidepressant, neuroprotective, analgesic, anti-inflammatory, muscle-relaxant and sedative/hypnotic properties^42^. Our results indicate that *Streptomyces* sp. VN1 is a promising producer of cinamamide compounds for drug discovery.

Lobophorin A and related analogs are members of the spirotetronate family. Their anti-inflammatory and antibacterial properties have been reported^16,43^. Genome sequence analysis results revealed that the putative lobophorin biosynthetic gene cluster in *Streptomyces* sp. VN1 showed very high similarity with *Streptomyces* sp. FXJ7.023 and *Streptomyces* sp. SCSIO 01127 (Fig. 3; Supplementary Table S5A). Four different analogs of lobophorin (compound **5**, **6**, **7** and **8**) were identified to be produced by *Streptomyces* sp. VN1 through mass analysis without any genetic modification. The diversity of lobophorin analogs has been previously explored. There has been a report of structural analysis of lobophorin revealing a polycyclic tetronolide core that is further modified by glycosylation and hydroxylation^35^. Through deletion of methyltransferase in *Streptomyces* sp. SCSIO 01127 the authors obtained two new analogs of lobophorin^39^. In addition, deletion of the glycosylation step in the biosynthesis pathway has achieved novel analogs of lobophorins^36^. The catalytic activity of core enzymes for lobophorin A biosynthesis and post-modification enzymes in *Streptomyces* sp. VN1 might be different from those in *Streptomyces* sp. SCSIO 01127. This might lead to different of lobophorin A analogs. In addition, different post-modification steps might convert intermediates to different analogs. Post-modification tailoring of lobophorin A might also follow a different succession leading to accumulation of novel analogs of lobophorin A in *Streptomyces* sp. VN1. The diversity of lobophorin analogs identified in *Streptomyces* sp. VN1 might be the result of flexibility of post-modification enzymes such as glycosyltransferase, methyltransferase and hydroxylase which might have different substrate preferences among intermediates of lobophorin. The presence of an N-acetyltransferase gene in cluster 1 of *Streptomyces* sp. VN1 might have played role in adding to the diversity of lobophorin analogs. The N-acetyltransferase gene might be responsible for transfer of an acetyl group to lobophorin A to form other derivatives such as compound **7** and compound **8** (Supplementary Table S5B). A similar analog of lobophorin has been reported in a previous study where lobophorin G is detected as acetylated lobophorin A^13^. The diversity of lobophorin analogs identified from *Streptomyces* sp. VN1 highlights the uniqueness of lobophorin biosynthetic pathway compared to other lobophorin producer strains. Thus, *Streptomyces* sp. VN1 can be developed into a prolific producer of lobophorin and its analogs can be further diversified through genetic engineering. Biosynthetic modifications such as glycosylation^44^, hydroxylation^45^ and methylation^46^ have been successfully applied for the biosynthesis of non-natural products. These modifications could be applied for *Streptomyces* sp. VN1 to produce novel lobophorin analogs with potential medical applications.

Cyclo-L-proline-L-tyrosine was first reported to be produced by *Streptomyces* sp. strain 22-4^17^. It had activities against three economically important plant pathogens, *Xanthomonas axonopodis* pv. citri, *Ralstonia solanacearum* and *Clavibacter michiganensis*.

Although the structure of compound **4** has been reported previously as a synthetic by-product (http://www.ambinter.com/search), unfortunately, no reference NMR data of compound **4** was available. To the best of our knowledge, this is the first report of NMR spectrum and biological activity of furan-type compound **4** produced by a marine *Streptomyces*. There has been a report of moderate production of a furan-type compound by microorganism and intermediate furan-type compound in *Streptomyces* that may function as a starting unit for a secondary metabolite, such as A-factor, butyrolactone, and other analogs of methylenomycin furans (MMFs)^47^ from *Streptomyces celicolor*.

In 2008, Jidong *et al*. reported a furan compound HS071 from *Streptomyces* sp. HS-HY-071 that showed cytotoxic activity against a cancer cell line^48^. However, there is no report about the biosynthesis pathway of this compound. There are some differences between structures of compound **4** and furan-compound HS071^48^, such as a 4-OH position in the HS071 compound where compound **4** contains a carboxyl group (Supplementary Fig. S16) probably because these two compounds are produced from two different biosynthetic pathways or because compound **4** or compound HS071 is further modified, although they come from the same biosynthesis pathway. The biosynthesis pathway of compound **4** was not explored in this study. Further studies by feeding ^13^C in combination with genome sequence data are needed to track down the exact mechanism of compound **4** biosynthesis.

We examined the biological activity of compound **4** against five different types of cancer cell lines. Compound **4** showed growth inhibitory activity most effectively against an AGS cancer cell line with an IC_50_ value of 40.5 µM. Although concentration ranges of compound **4** for growth inhibition of cancer cells were much higher than those of known anticancer drugs such as taxol^49^ with effective concentrations below 10 µM, compound **4** inhibited the growth of cancer cell lines more effectively as compared to normal cell lines at tested concentrations (Fig. 7). Interestingly, compound **4** also showed anti-migration and anti-invasion activities against AGS cancer cell line after 24 hours of exposure (Figs. 8 and 9). These results confirm compound **4** possesses anticancer activity. However, as shown in cell growth assay the cell viability was high (89%) even after treatment with compound **4** at 25 µM treatment for 72 hours. Therefore, inhibitory effects of compound **4** on the migration and invasion of AGS cancer cells were not merely due to the cytotoxicity of compound **4**. Such anti-invasive properties of compound **4** against cancer cell lines showcase the relevance of exploring secondary metabolites biosynthetic pathways for discovering clinically relevant compounds.

Results of *in sillico* genome analysis showed that *Streptomyces* sp. VN1 encodes the putative biosynthetic gene clusters of diverse interesting secondary metabolites, such as carotenoid, friulimicin, xiamycin, divergolide, and informatipeptin. However, biosynthetic pathways for these metabolites in *Streptomyces* sp. VN1 were inactive under normal culture conditions. This study displays the biosynthetic potential of *Streptomyces* sp. VN1 through isolation and characterization of four compounds. Due to its ability to grow fast and produce various secondary metabolites, it is suitable for further metabolic exploration to produce useful metabolites. We present *Streptomyces* sp. VN1 as a good candidate for strain optimization to produce therapeutically and industrially relevant compounds. Our results reinforce the need of further exploring marine *Streptomycetes* as a rich source of novel metabolites relevant for biotechnological applications.

## Supplementary information

Supplementary data.
