# Supplementary material for: Streptomyces sp. VN1, a producer of diverse metabolites including non-natural furan-type anticancer compound
Source: Sci Rep. 2020 Feb 4;10:1756. doi: 10.1038/s41598-020-58623-1 (PMC7000394; doi:10.1038/s41598-020-58623-1)
Supplement: Supplementary file 1 — Supplementary data. [file 41598_2020_58623_MOESM1_ESM.docx]

Scientific Reports

Supplementary information

***Streptomyces* sp. VN1, a producer of diverse metabolites including non-natural furan-type anticancer compound**

Hue Thi Nguyen^1^, Anaya Raj Pokhrel^1^, Chung Thanh Nguyen^1^, Van Thuy Thi Pham^1^, Dipesh Dhakal^1^, Haet Nim Lim^1^, Hye Jin Jung^1,2^, Tae-Su Kim^1^, Tokutaro Yamaguchi^1,2^, Jae Kyung Sohng^1,2,*^

*^1^Department of Life Science and Biochemical Engineering, SunMoon University, 70 Sunmoon-ro 221, Tangjeong-myeon, Asan-si, Chungnam 31460, Republic of Korea*

*^2^Department of Pharmaceutical Engineering and Biotechnology, SunMoon University, 70 Sunmoon-ro 221, Tangjeong-myeon, Asan-si, Chungnam 31460, Republic of Korea.*

***Corresponding author:** Prof. Jae Kyung Sohng

Tel: +82(41)530-2246

Email: sohng@sunmoon.ac.kr

Fax: +82(41)530-8229

**Table lists**

**Table S1.** Characteristics of the strain by observation of growth on nutrients and salt constituents

**Table S2.** Major fatty acid composition of *Streptomyces* sp. VN1 represented in percentage. Standard FAME nomenclature has been used where number after colon indicates number of double bonds in the molecule and the carbon numbering begins from aliphatic ends.

**Table S3.** Genome-to-Genome Distance and *in silico* DNA-DNA hybridization of *Streptomyces* sp. VN1

**Table S4.** Overview of 34 secondary metabolites of biosynthetic gene clusters analysis by antiSMASH. The number of BGCs determined with antiSMASH with ClusterFinder OFF.

**Table S5.** The comparison of the cluster 1 which annotated for oligosaccharide-T1PKS-T3PKS-NRPS biosynthesis.

(**A**) The comparison of the lobophorin gene clusters. ^a^lobophorin gene cluster from *Streptomyces* sp. VN1, ^b^lobophorin gene cluster from *Streptomyces olivaceus* FXJ7.023 (GenBank: JX306680); ^c^lobophorin gene cluster from *Streptomyces* sp. SCSIO 01127 (GenBank: KC013978). aa: amino acids, (**B**) The comparison of the totopotensamides gene clusters. ^a^ totopotensamides gene cluster from *Streptomyces* sp. VN1; ^b^totopotensamides gene cluster from *Streptomyces* sp. SCSIO 02999 (GenBank: MG012231). aa: amino acids.

**Table S6.** (**A**) ^1^H- and ^13^C-NMR data of cinnamamide (**1**) in methanol-*d_4_*, (**B**) ^1^H- and ^13^C-NMR data of lobophorin A (**2**) in methanol-*d_4_*, (**C**) ^1^H- and ^13^C-NMR data of cyclo-L-proline-L-tyrosine (**3**) in methanol-*d_4_*.

**Figure lists**

**Figure S1.** EggNOG functional classification of proteins in the *Streptomyces* sp. VN1 genome. The distribution of the predicted proteins was assigned by the COG database.

**Figure S2.** Proposed organization biosynthetic gene cluster of carotenoid-like compound in the Streptomyces sp. VN1. The highest similarity gene cluster from *Streptomyces avermitilis* is shown, corresponding genes was highlight in same color.

**Figure S3.** Proposed organization of biosynthetic gene cluster of friulimicin-analog compound in the Streptomyces sp. VN1. The highest similarity gene cluster from *Actinoplanes friuliensis* is shown, corresponding genes was highlight in same color.

**Figure S4.** Proposed organization of biosynthetic gene cluster of xiamycin-derivative compound in the Streptomyces sp. VN1. The highest similarity gene cluster from *Streptomyces* sp. SCSIO 02999 is shown, corresponding genes was highlight in same color.

**Figure S5.** Proposed organization of biosynthetic gene cluster of enterocin in the Streptomyces sp. VN1. The highest similarity gene cluster from *Streptomyces maritimus* is shown, corresponding genes was highlight in same color.

**Figure S6.** Proposed organization biosynthetic gene cluster of divergolide compound in the Streptomyces sp. VN1. The most similar gene cluster from *Streptomyces* sp. HKI0576 is shown, corresponding genes was highlight in same color.

**Figure S7.** Proposed organization biosynthetic gene cluster of informatipeptin-like compound in the Streptomyces sp. VN1. The highest similarity gene cluster from *Streptomyces viridochromogenes* DSM 40736 is shown, corresponding genes was highlight in same color.

**Figure S8.** Proposed organization biosynthetic gene cluster of herboxidiene-like compound in the Streptomyces sp. VN1. The highest similarity gene cluster from *Streptomyces chromofuscus* A7847 is shown, corresponding genes was highlight in same color.

**Figure S9.** Proposed organization of biosynthetic gene cluster of nogalamycin-like compound in the Streptomyces sp. VN1. The highest similarity gene cluster from *Streptomyces nogalater* is shown, corresponding genes was highlight in same color.

**Figure S10.** (**A**) HR-Q-TOF-MS, MS/MS of compound **1**, (**B**) HR-Q-TOF-MS of compound **2**, (**C**) HR-Q-TOF-MS, MS/MS of compound **3**.

**Figure S11.** HR-Q-TOF-MS, MS/MS of compound **4**.

**Figure S12.** IR data of compound **4**.

**Figure S13.** NMR data of compound **4**. (**A**) ^1^H-NMR, (**B**) ^13^C-NMR, (**C**) COSY-NMR, (**D**) ROESY-NMR, (**E**) HSQC-DEPT and (**F**) HMBC data of compound **4**.

**Figure S14.** The CD spectrum of compound **4**. CD (solid lines) with the associated HT (high tension) (dashed lines) spectrum which is related to the absorbance of the sample.

**Figure S15.** (**A**) Propose fragmentation of lobophorin A, (**B**) HR-Q-TOF-MS/MS profile of lobophorin A, (**C**) HR-Q-TOF-MS/MS profile of compound **5**, (**D**) HR-Q-TOF-MS/MS profile of compound **6**, (**E**) HR-Q-TOF-MS/MS profile of compound **7** and (**F**) HR-Q-TOF-MS/MS profile of compound **8**.

**Figure S16.** Structures of compound **4** and compound HS071

**Table S1**

| **Characteristics** | ***Streptomyces* sp. VN1** |
| --- | --- |
| **Growth on nutrients** |  |
| Yeast Extract | + |
| Malt Extract | + |
| TES | + |
| Beef Extract | + |
| Oat Meal | + |
| **Growth on salt constituents** |  |
| MgCl_2_.6H_2_O | + |
| K_2_SO_4_ | + |
| KH_2_PO_4_ (0.5%) | + |
| CaCl_2_.2H_2_O (3.68%) | + |
| NaCl (3%) | + |
| FeSO_4_ (0.05%) | + |
| MgSO_4_ (0.05%) | + |
| KH_2_PO_4_ (0.05%) | + |
| MnSO_4_ | - |
| NH_4_(NO_3_)_2_ | - |
| KCl | + |
| NaNO_3_ | - |
| CaNO_3_ | - |
| CaCO_3_ | + |
| Na_2_SO_4_ | - |

**Table S2.**

| **Fatty acid composition** | **Percentage composition** |
| --- | --- |
| C13:0 iso | 0.40 |
| C13:0 anteiso | 0.51 |
| C14:0 iso | 6.45 |
| C14:0 | 1.02 |
| C15:0 iso | 10.29 |
| C15:0 anteiso | 19.41 |
| C15:0 | 1.11 |
| C16:1 iso H | 3.31 |
| C16:0 iso | 23.61 |
| C16:1 cis 9 | 7.57 |
| C16:0 | 11.09 |
| C16:0 9 Methyl | 2.76 |
| C17:1 anteiso C | 2.16 |
| C17:0 iso | 3.97 |
| C17:0 anteiso | 7.23 |
| C17:0 cyclo | 2.94 |

**Table S3.**

| **Query genome** | **Reference genome** | **Model Calculate** | **Bootstrap Calculate** | | **Distance** | **Probe DDH** | **G+C difference (>= 70%)** |
| --- | --- | --- | --- | --- | --- | --- | --- |
| *Streptomyces* sp. VN1 | *Streptomyces* sp. FXJ7.023 (GCF_000404005.1) | [94.1 - 97.1%] | | 95.9 - 95.9% | 0.0658 | 99.87 | 0.04 |
| *Streptomyces* sp. VN1 | *Streptomyces pactum* (GCF_001767375.1) | [90.6 - 94.7%] | | 93 - 93% | 0.089 | 99.71 | 0.03 |
| *Streptomyces* sp. VN1 | *Streptomyces olivaceus* (GCF_000721235.1) | [89.1 - 93.6%] | | 91.6 - 91.6% | 0.0986 | 99.6 | 0.02 |

**Table S4.**

| Cluster | Type | From | To | Most similar known biosynthetic gene cluster (percent of similarity) | Reference strain | Accession number |
| --- | --- | --- | --- | --- | --- | --- |
| 1 | OligosaccharT3PKS-NRPS | 10491 | 225803 | Lobophorin A (96%) | *Streptomyces* sp. FXJ7.023 | JX306680 |
| 2 | Terpene | 237497 | 258555 | 2-methylisoborneol (100%) | *Streptomyces griseus* NBRC 13350 | AP009493 |
| 3 | T3PKS | 280535 | 321779 | Herboxidiene (2%) | *Streptomyces chromofuscus* A7847 | JN671974 |
| 4 | Indole | 467588 | 488715 | Antimycin (20%) | *Streptomyces* sp. S4 | NZ_FR873698 |
| 5 | Terpene | 539486 | 563714 | Carotenoid (54%) | *Streptomyces avermitilis* | AB070934 |
| 6 | Amglyccycl | 741943 | 763181 | Validamycin (22%) | *Streptomyces hygroscopicus* | DQ223652 |
| 7 | T3PKS | 992279 | 1033391 | Herboxidiene (8%) | *Streptomyces chromofuscus* A7847 | JN671974 |
| 8 | NRPS | 1105368 | 1158770 | Kirromycin (5%) | *Streptomyces collinus* Tu 365 | AM746336 |
| 9 | Ectoine | 1684875 | 1695273 | Ectoine (100%) | *Streptomyces anulatus* | AY524544 |
| 10 | Melanin | 2650272 | 2660898 | Melanin (100%) | *Streptomyces coelicolor* A3(2) | AL645882 |
| 11 | Lassopeptide | 2720839 | 2743360 | SSV-2083 (50%) | *Streptomyces sviceus* ATCC 29083 | NZ_CM000951 |
| 12 | Siderophore | 2756614 | 2768407 | Desferrioxamine_B (83%) | *Streptomyces coelicolor A3(2)* | AL645882 |
| 13 | Phenazine | 3460126 | 3480566 |  |  |  |
| 14 | Thiopeptide | 3704677 | 3730907 | Diazepinomicin (7%) | *Micromonospora* sp. M42 | KK037233 |
| 15 | Lantipeptide | 4240796 | 4272172 | SBI-06990 alpha/SBI-06989 beta (50%) | *Streptomyces bingchenggensis* BCW-1 | CP002047 |
| 16 | NRPS | 4547286 | 4588689 | Phosphonoglycans (3%) | *Glycomyces* sp. NRRL B-16210 | KJ125437 |
| 17 | Terpene | 5321623 | 5342708 | Albaflavenone B (100%) | *Streptomyces coelicolor* A3(2) | AL645882 |
| 18 | T2PKS | 5408372 | 5450926 | Spore_pigment (66%) | *Streptomyces avermitilis* | AB070937 |
| 19 | Siderophore | 5952135 | 5964267 |  |  |  |
| 20 | T1PKS-NRPS | 6048796 | 6212376 | Friulimicin (75%) | *Actinoplanes friuliensis* | AJ488769 |
| 21 | T1PKS-NRPS | 6260144 | 6309491 | Xiamycin (77%) | *Streptomyces* sp. SCSIO 02999 | JQ812811 |
| 22 | Bacteriocin | 6350672 | 6362015 |  |  |  |
| 23 | Terpene | 6392352 | 6414562 |  |  |  |
| 24 | Siderophore | 6562337 | 6575511 |  |  |  |
| 25 | NRPS | 6603510 | 6649936 | Herbimycin (13%) | *Streptomyces hygroscopicus* AM3672 | AY947889 |
| 26 | T2PKS | 6650019 | 6692435 | Enterocin (95%) | *Streptomyces maritimus* | AF254925 |
| 27 | Lantipeptide | 6764643 | 6800414 |  |  |  |
| 28 | Nucleoside-NRPS | 6830998 | 6877942 | Nogalamycin (40%) | *Streptomyces nogalater* | AF323753 |
| 29 | Terpene-NRPS | 7137719 | 7221298 | Hopene (92%) | *Streptomyces coelicolor* A3(2) | AL645882 |
| 30 | T1PKS | 7275849 | 7358369 | Divergolide (100%) | *Streptomyces* sp. HKI0576 | HF563079 |
| 31 | Terpene | 7656962 | 7677990 | Versipelostatin (5%) | *Streptomyces versipellis* 4083-SVS6 | LC006086 |
| 32 | Bacteriocin | 7687326 | 7697541 | Informatipeptin (42%) | *Streptomyces viridochromogenes* DSM 40736 | GG657757 |
| 33 | NRPS | 7898917 | 7949836 | Coelichelin (100%) | *Streptomyces coelicolor* A3(2) | AL645882 |
| 34 | T1PKS | 8088142 | 8176725 | Rifamycin (9%) | *Amycolatopsis mediterranei* S699 | AF040570 |

**Table S5.**

(**A**)

| **Gene^a^** | **Size**  **(aa)^*^** | **Gene^b^** | **Accession number, identity/similarity %)** | **Gene^c^** | **Accession number, identity/similarity %)** | **Proposed function** |
| --- | --- | --- | --- | --- | --- | --- |
| *orf* 10 | 100 |  |  |  |  | Hypothetical protein |
| *orf* 11 | 131 |  |  |  |  | Hypothetical protein |
| *orf* 12 | 291 |  |  |  |  |  |
| *orf* 13 | 914 | *luxR* | AGC09469.1, 99/98 |  |  | LuxR family transcriptional regulator |
| *orf* 14 | 430 |  | AGC09470.1, 97/97 |  |  | MATE efflux family protein |
| *orf* 15 | 160 |  | AGC09471.1, 100/100 |  |  | Chloramphenicol acetyltransferase |
| *orf* 16 | 309 |  |  | *lobR5* | AGI99509.1, 98/98 | LysR family transcriptional regulator |
| *orf* 17 | 943 |  | AGC09472.1, 91/91 |  |  | Cotonyl-CoA reductase/alcohol behydrogenase. |
| *orf* 18 | 128 |  | AGC09474.1, 98/98 |  |  | Putative protein |
| *orf* 19 | 274 | *lobR1* | AGC09475.1, 99/100 | *lobR3* | AGI99506.1, 99/99 | Transcriptional regulator, SARP family |
| *orf* 20 | 202 | *lobD8* | AGC09476.1, 99/100 | *lobS11* | AGI99505.1, 99/100 | Sugar 5-epimerase |
| *orf* 21 | 332 | *lobD7* | AGC09477.1, 100/100 | *lobS10* | AGI99504.1, 99/99 | Oxidoreductase |
| *orf* 22 | 298 | *lobD6* | AGC09478.1, 99/99 | *lobS9* | AGI99503.1, 99/99 | G-1-P adenylyl/thymidylyltransferase |
| *orf* 23 | 344 | *lobD5* | AGC09479.1, 99/99 | *lobS8* | AGI99502.1, 99/99 | NAD-dependent epimerase/dehydratase |
| *orf* 24 | 439 | *lobD4* | AGC09480.1, 99/99 | *lobS7* | AGI99501.1, 99/100 | Acyl-CoA dehydrogenase |
| *orf* 25 | 373 | *lobD3* | AGC09481.1, 99/99 | *lobS6* | AGI99500.1, 99/99 | Sugar 3-aminotransferase |
| *orf* 26 | 414 | *lobD2* | AGC09482.1, 99/99 | *lobS7* | AGI99499.1, 98/98 | Methyltransferase |
| *orf* 27 | 133 | *lobD1* | AGC09483.1, 98/98 | *lonU2* | AGI99498.1, 97/98 | Unknown |
| *orf* 28 | 6347 | *lobS1* | AGC09484.1, 99/99 | *lobA5* | AGI99497.1, 97/97 | Type I polyketide synthase |
| *orf* 29 | 7169 | *lobS2* | AGC09485.1, 97/97 | *lobA4* | AGI99496.1, 97/97 | Type I polyketide synthase |
| *orf* 30 | 1797 | *lobS3* | AGC09486.1, 87/89 | *lobA3* | AGI99495.1, 95/96 | Type I polyketide synthase |
| *orf* 31 | 1564 | *lobS5* | AGC09487.1, 97/97 | *lobA2* | AGI99494.1, 96/96 | Type I polyketide synthase |
| *orf* 32 | 492 | *lobA* | AGC09488.1, 99/99 | *lobP3* | AGI99493.1, 99/100 | FAD-dependent oxidoreductase |
| *orf* 33 | 342 | *lobB* | AGC09489.1, 99/99 | *lobC4* | AGI99492.1, 99/99 | Ketoacyl acylcarrier protein synthase III |
| *orf* 34 | 621 | *lobC* | AGC09490.1, 99/99 | *lobC3* | AGI99491.1, 99/99 | FkbH-like protein (acetyl-transferase) |
| *orf* 35 | 75 | *lobC0* | AGC09491.1, 99/100 | *lobC2* | AGI99490.1, 99/100 | Acyl carrier protein |
| *orf* 36 | 616 | *lobE* | AGC09492.1, 99/99 | *lobC1* | AGI99489.1, 98/99 | Hydrolase superfamily dihydrolipoamide |
| *orf* 37 | 211 | *lobR2* | AGC09493.1, 99/100 | *lobR2* | AGI99488.1, 98/99 | TetR family transcriptional regulator |
| *orf* 38 | 400 | *lobC4* | AGC09494.1, 99/100 | *lobG3* | AGI99487.1, 99/99 | Glycosyltransferase MGT family |
| *orf* 39 | 416 | *lobC3* | AGC09495.1, 99/99 | *lobG2* | AGI99486.1, 98/99 | Glycosyltransferase MGT family |
| *orf* 40 | 506 | *lobB3* | AGC09496.1, 100/100 | *lobP2* | AGI99485.1, 98/99 | FAD-dependent oxidoreductase |
| *orf* 41 | 240 | *lobB2* | AGC09497.1, 100/100 | *lobB* | AGI99484.1, 99/99 | Thioesterase |
| *orf* 42 | 483 | *lobB1* | AGC09498.1, 99/100 | *lobS4* | AGC09498.1, 98/98 | Sugar 2,3-dehydratase |
| *orf* 43 | 3936 | *lobS4* | AGC09499.1, 94/94 | *lobA1* | AGI99482.1, 98/98 | Type I polyketide synthase |
| *orf* 44 | 391 | *lobA0* | AGC09500.1, 99/100 | *lobG1* | AGI99481.1, 98/99 | Glycosyltransferase MGT family |
| *orf* 45 | 266 | *lobA9* | AGC09501.1, 100/100 | *lobS3* | AGI99480.1, 99/99 | SAM-dependent methyltransferase |
| *orf* 46 | 384 | *lobA8* | AGC09502.1, 100/100 | *lobS2* | AGI99479.1, 98/98 | Aminotransferase |
| *orf* 47 | 272 | *lobA7* | AGC09503.1, 99/99 | *lobS1* | AGI99478.1, 99/99 | Sugar-O-methyltransferase |
| *orf* 48 | 326 | *lobA6* | AGC09504.1, 99/99 | *lobU1* | AGI99477.1, 99/99 | Aldo/keto reductase |
| *orf* 49 | 392 | *lobA5* | AGC09505.1, 99/99 | *lobP1* | AGI99476.1, 99/99 | p450 monooxygenase |
| *orf* 50 | 497 | *lobA4* | AGC09506.1, 100/100 | *lobT1* | AGI99475.1. 99/99 | Efflux permease |
| *orf* 51 | 195 | *lobR3* | AGC09507.1, 100/100 | *lobR1* | AGI99474.1, 96/96 | TetR type regulatory protein |
| *orf* 52 | 260 | *lobA3* | AGC09508.1, 99/100 |  |  | FkbM family methyltransferase |

(**B**)

| **Gene^a^** | **Size**  **(aa)^*^** | **Gene^b^** | **Accession number, identity/similarity %)** | **Proposed function** |
| --- | --- | --- | --- | --- |
| *orf* 51 | 195 | *totR5* | ATL73059.1, 96/96 | TetR family transcriptional regulator |
| *orf* 52 | 260 | *totU5* | ATL73058.1, 96/98 | FkbM family methyltransferase |
| *orf* 53 | 393 | *totG* | ATL73057.1, 98/98 | Glycosyltransferase MGT family |
| *orf* 54 | 898 | *totR4* | ATL73056.1, 98/98 | LuxR family transcriptional regulator |
| *orf* 55 | 744 | *totD2* | ATL73055.1, 98/98 | Cysteine synthase |
| *orf* 56 | 71 | *totI* | ATL73054.1, 99/100 | mbtH-like protein |
| *orf* 57 | 208 | *totR3* | ATL73053.1, 99/100 | TetR family transcriptional regulator |
| *orf* 58 | 913 | *totR2* | ATL73052.1, 98/99 | LuxR family transcriptional regulator |
| *orf* 59 | 881 | *totR1* | ATL73051.1, 97/97 | LuxR family transcriptional regulator |
| *orf* 60 | 123 | *totU4* | ATL73050.1, 98/98 | ketosteroid isomerase-like protein |
| *orf* 61 | 480 | *totH* | ATL73049.1. 98/98 | FAD dependent halogenase |
| *orf* 62 | 305 | *totK* | ATL73048.1, 99/99 | Hypothetical protein |
| *orf* 63 | 495 | *totD1* | ATL73047.1, 99/99 | Argininosuccinate lyase/adenylosuccinate lyase |
| *orf* 64 | 544 | *totB3* | ATL73046.1, 97/96 | Hybrid PKS-NRPS protein |
| *orf* 65 | 1079 | *totB2* | ATL73045.1, 98/98 | AMP-dependent synthetase/ligase |
| *orf* 66 | 382 | *totE2* | ATL73044.1, 99/99 | *scyllo*-Inosamine-4-phosphate amidinotransferase |
| *orf* 67 | 434 | *totC4* | ATL73043.1, 99/99 | Aminotransferase |
| *orf* 68 | 459 | *totC3* | ATL73042.1, 97/97 | Enoyl-CoA-hydratase |
| *orf* 69 | 238 | *totC2* | ATL73041.1, 99/98 | Enoyl-CoA-hydratase |
| *orf* 70 | 373 | *totC1* | ATL73040.1,99/99 | Chalcone/stilbene synthase domain protein |
| *orf* 71 | 303 | *totP4* | ATL73039.1, 99/99 | Taurine dioxygenase |
| *orf* 72 | 304 | *totS 303* | ATL73038.1, 99/99 | Glycolipid sulfotransferase |
| *orf* 73 | 405 | *totP3* | ATL73037.1, 99/100 | Cytochrome P450 hydroxylase |
| *orf* 74 | 6243 | *totB1* | ATL73036.1, 98/98 | Amino acid adenylation protein |
| *orf* 75 | 355 | *TotM* | ATL73035.1, 99/100 | O-methyltransferase family protein |
| *orf* 76 | 3736 | *totA2* | ATL73034.1, 97/98 | Type I modular polyketide synthase |
| *orf* 77 | 6526 | *totA1* | ATL73033.1, 98/98 | Type I modular polyketide synthase |
| *orf* 78 | 383 | *totE1* | ATL73032.1, 99/99 | *scyllo*-Inosamine-4-phosphate amidinotransferase |
| *orf* 79 | 401 | *totP2* | ATL73031.1, 99/99 | Cytochrome P450 hydroxylase |
| *orf* 80 | 80 | *totF* | ATL73030.1, 97/100 | Ferredoxin |
| *orf* 81 | 82 | ? | ? |  |
| *orf* 82 | 230 | *totP1* | ATL73029.1, 98/97 | Luciferase-like monooxygenase |
| *orf* 83 | 95 | *touU3* | ATL73028.1, 98/98 | Hypothetical protein |
| *orf* 84 | 191 | *totU2* | ATL73027.1, 98/98 | Secreted extracellular small neutral protease |
| *orf* 85 | 299 | *totU1* | ATL73026.1, 96/97 | Virginiamycin B lyase |
| *orf* 86 | 379 | *orf(-3)* | ATL73025.1, 96/97 | Oxidoreductase |
| *orf* 87 | 282 | *orf(-2 )* | ATL73024.1, 99/99 | Transcriptional regulator |
| *orf* 88 | 290 | *orf (-1)* | ATL73023.1, 99/99 | ABC-type transport protein |
| *orf* 89 | 351 | ? | ? | ABC transporter substrate-binding protein |
| *orf* 90 | 299 | ? | ? | ABC transporter permease subunit |
| *orf* 91 | 276 | ? | ? | ABC transporter ATP-binding protein |
| *orf* 92 | 301 | ? | ? | Nitrate ABC transporter substrate-binding protein |
| *orf* 93 | 400 | ? | ? | Major facilitator transporter |
| *orf* 94 | 371 | ? | ? | Iron compound ABC transporter, periplasmic |
| *orf* 95 | 337 | ? | ? | ABC transporter ATP-binding protein |
| *orf* 96 | 367 | ? | ? | Transport system permease protein |
| *orf* 97 | 210 | ? | ? | TetR family transcriptional regulator |
| *orf* 98 | 183 | ? | ? | N-acetyltransferase |

**Table S6.**

(**A**)

| **No** | **^13^C (ppm)** | **^1^H (ppm)** |
| --- | --- | --- |
| 1 | 134.79 |  |
| 2 | 127.51 | 7.59 (dd, *J*=8.0, 1.6) |
| 3 | 128.57 | 7.41 (dd, *J* = 7.1, 7.1) |
| 4 | 129.55 | 7.39 (dddd, J = 6.9, 6.9, 1.3, 1.3) |
| 5 | 128.57 | 7.41 (dd, *J* = 7.1, 7.1) |
| 6 | 127.51 | 7.59 (dd, *J*=8.0, 1.6) |
| 7 | 141.31 | 7.57 (d, *J* = 15.5) |
| 8 | 120.02 | 6.66 (d, *J* = 15.6) |
| 9 | 169.54 |  |

(**B**)

| **No** | **^13^C (ppm)** | **^1^H (ppm)** |  | **No** | **^13^C (ppm)** | **^1^H (ppm)** |
| --- | --- | --- | --- | --- | --- | --- |
| D1 C | 96.18 | 4.75 (d, *J* = 10.1) |  | 1 C | 168.71 |  |
| D2 C | 36.27 | 1.86 (d, *J* = 14.9) |  | 2 C | 102.03 |  |
|  |  | 1.94 (d, *J* = 14.9) |  | 3 C | 204.42 |  |
| D3 C | 56.71 |  |  | 4 C | 51.01 |  |
| D4 C | 53.87 | 3.64 (d, *J* = 9.6) |  | 5 C | 43.38 | 2.09 (dd, *J* = 6.7, 2.6) |
| D5 C | 67.48 | 3.99 (dq, *J* = 6.5, 6.5) |  | 6 C | 31.09 | 1.66 – 1.61 (m) |
| D6 C | 15.96 | 1.19 (d, *J* = 6.1) |  | 7 C | 41.53 | 1.58 – 1.54 (m) |
| D7 C | 22.87 | 1.37 (s) |  |  |  | 1.66 – 1.61 (m) |
| D8 N |  | 7.34 (dd, *J* = 10.1, 2.6) |  | 8 C | 34.51 | 2.29 – 2.23 (m) |
| D9 C | 158.62 |  |  | 9 C | 84.59 | 3.44 (dd, *J* = 10.6, 5.2) |
| D10 C | 51.64 | 3.70 (s) |  | 10 C | 38.28 | 2.16 – 2.10 (m) |
|  |  |  |  | 11 C | 126.03 | 5.84 (d, *J* = 10.2) |
| A1 C | 98.31 | 4.77 (d, *J* = 4.3) |  | 12 C | 126.17 | 5.38 (ddd, *J* = 10.4, 5.0, 2.4) |
| A2 C | 29.62 | 1.78 – 1.73 (m) |  | 13 C | 52.92 | 3.54 – 3.50 (m) |
|  |  | 2.38 (d, *J* = 15.1) |  | 14 C | 135.84 |  |
| A3 C | 67.82 | 4.03 (ddd, *J* = 3.2, 3.2, 3.2) |  | 15 C | 123.42 | 5.21 (d, *J* = 9.5) |
| A4 C | 71.87 | 3.30 (dd, *J* = 9.6, 3.0) |  | 16 C | 30.94 | 2.30 (dd, *J* = 12.7, 5.8) |
| A5 C | 64.58 | 4.15 – 4.09 (m) |  |  |  | 2.48 – 2.42 (m) |
| A6 C | 16.87 | 1.23 (d, *J* = 3.2) |  | 17 C | 78.64 | 4.35 – 4.31 (m) |
|  |  |  |  | 18 C | 137.94 |  |
| B1 C | 91.74 | 5.18 (d, *J* = 3.9) |  | 19 C | 118.51 | 5.13 (d, *J* = 10.6) |
| B2 C | 34.51 | 2.01 (ddd, *J* = 14.6, 3.9, 3.9) |  | 20 C | 40.03 | 3.68 – 3.65 (m) |
|  |  | 2.12 – 2.08 (m) |  | 21 C | 120.60 | 5.46 (s) |
| B3 C | 66.64 | 4.21 (dd, *J* = 3.4, 3.4) |  | 22 C | 141.20 |  |
| B4 C | 81.91 | 3.30 (dd, *J* = 9.6, 3.0) |  | 23 C | 27.61 | 2.65 (dqd, *J* = 7.3, 6.7, 1.4) |
| B5 C | 62.25 | 4.08 (dq, *J* = 9.4, 6.0) |  | 24 C | 34.90 | 1.87 – 1.82 (m) |
| B6 C | 16.67 | 1.22 (d, *J* = 3.1) |  |  |  | 2.44 – 2.38 (m) |
|  |  |  |  | 25 C | 83.41 |  |
| C1 C | 99.33 | 4.97 (dd, *J* = 9.7, 2.1) |  | 26 C | 198.99 |  |
| C2 C | 37.39 | 1.77 – 1.70 (m) |  | 27 C | 14.14 | 1.59 (s) |
|  |  | 2.09 – 2.04 (m) |  | 28 C | 21.51 | 0.67 (d, *J* = 5.1) |
| C3 C | 63.01 | 4.31 (ddd, *J* = 3.2, 3.2, 3.2) |  | 29 C | 13.39 | 1.16 (d, *J* = 6.2) |
| C4 C | 82.38 | 2.87 (dd, *J* = 9.4, 2.8) |  | 30 C | 12.73 | 1.40 (s) |
| C5 C | 68.26 | 3.83 (dq, *J* = 9.4, 6.2) |  | 31 C | 13.86 | 1.45 (s) |
| C6 C | 17.29 | 1.25 (d, *J* = 6.2) |  | 32 C | 63.49 | 4.13 – 4.09 (m) |
| C7 C | 55.64 | 3.40 (s) |  |  |  | 4.19 (d, *J* = 14.5) |
|  |  |  |  | 33 C | 19.10 | 1.29 (d, *J* = 7.3) |

(**C**)

| **No** | **^13^C (ppm)** | **^1^H (ppm)** |
| --- | --- | --- |
| 1 |  |  |
| 2 | 165.56 |  |
| 3 | 56.51 | 4.38 (ddd, *J* = 5.0, 2.1) |
| 4 |  |  |
| 5 | 169.36 |  |
| 6 | 58.66 | 4.07 (ddd, *J* =10.8, 6.2, 1.9) |
| 7 | 28 | 2.15-2.09 (m)  1.24 (dddd, *J* = 12.1, 10.5, 10.1, 9.1) |
| 8 | 21.32 | 1.83 (m) |
| 9 | 44.52 | 3.57 (ddd, *J* = 11.9, 8.3)  3.37 (ddd, *J* =12.5, 6.5) |
| 10 | 36.29 | 3.11 (dd, *J* =14.2, 5.2)  3.05 (dd, *J* =14.2, 4.6) |
| 11 | 126.21 |  |
| 12 | 130.72 | 7.06 (d, *J* = 8.5) |
| 13 | 114.78 | 6.72 (d, *J* = 8.5) |
| 14 | 156.28 |  |
| 15 | 114.78 | 6.72 (d, *J* = 8.5) |
| 16 | 130.72 | 7.06 (d, *J* = 8.5) |

**Figure S1**


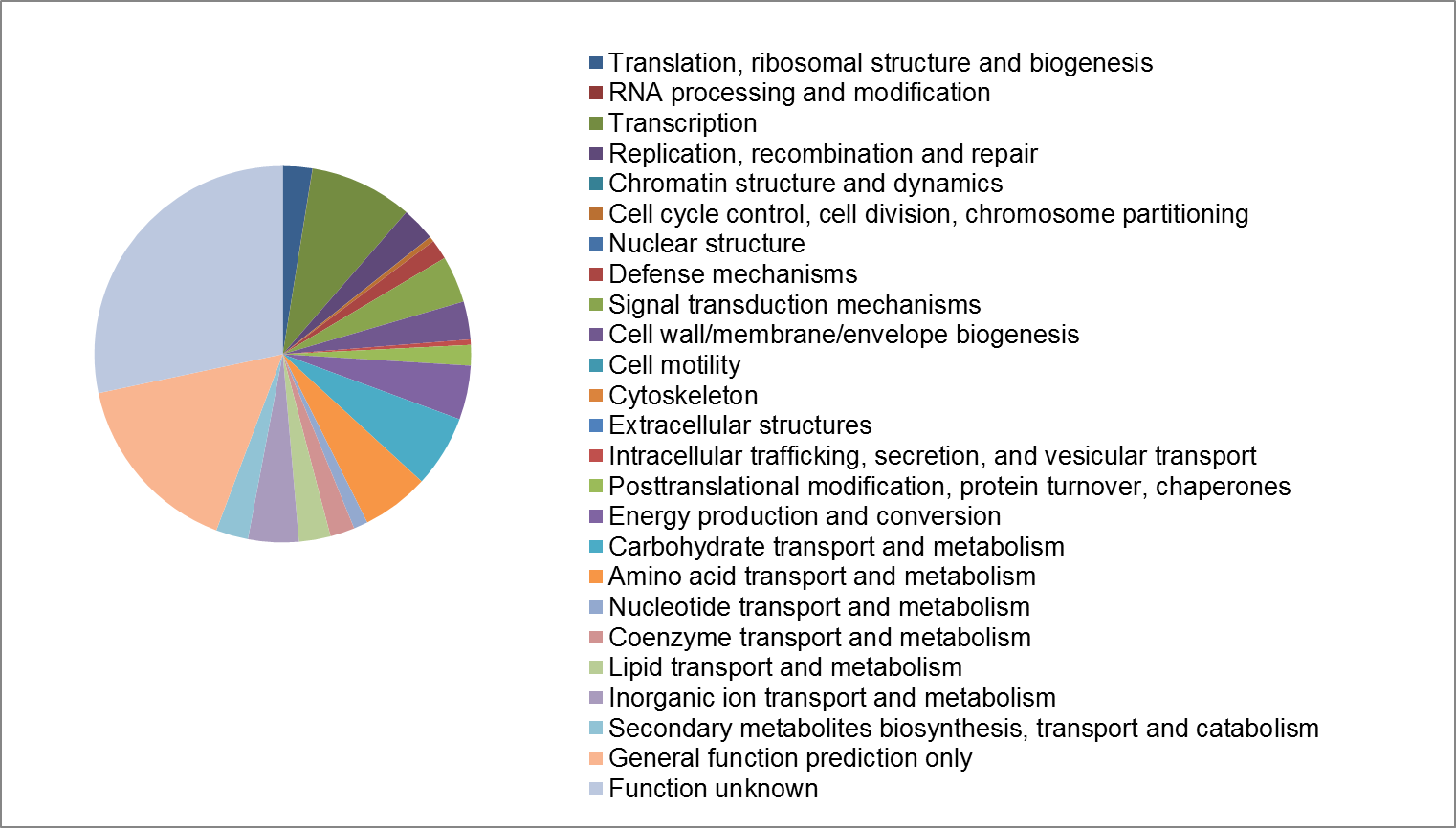


**Figure S2.**

**
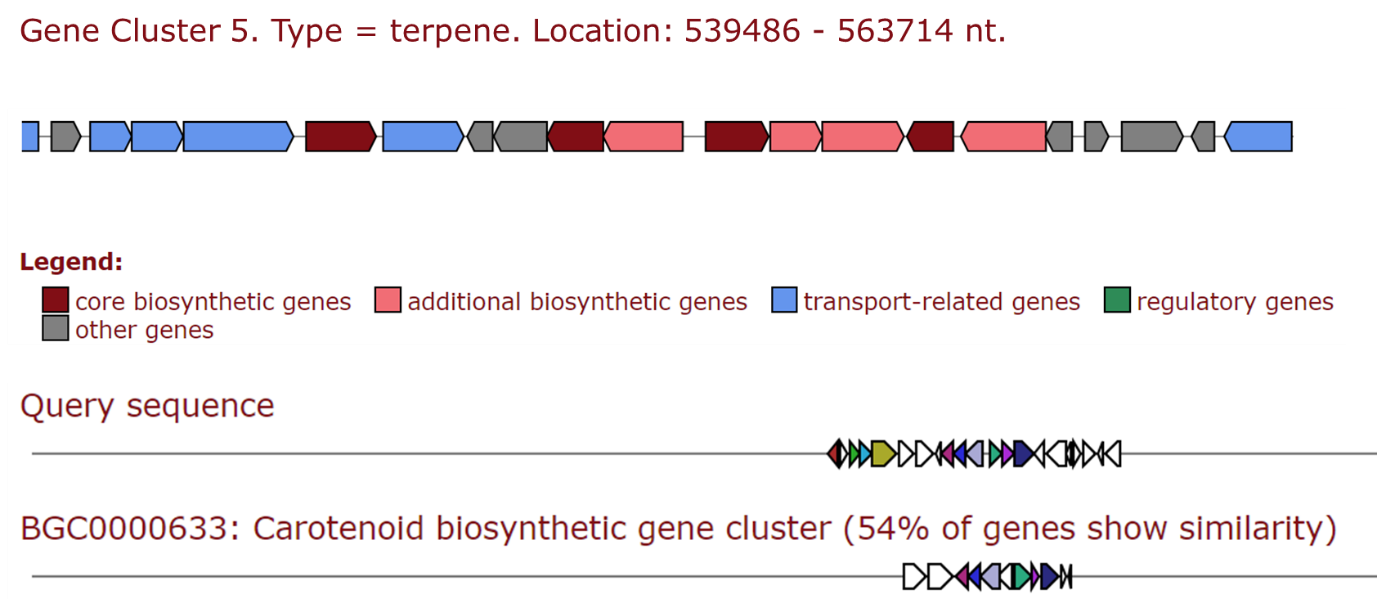
**

**Figure S3.**


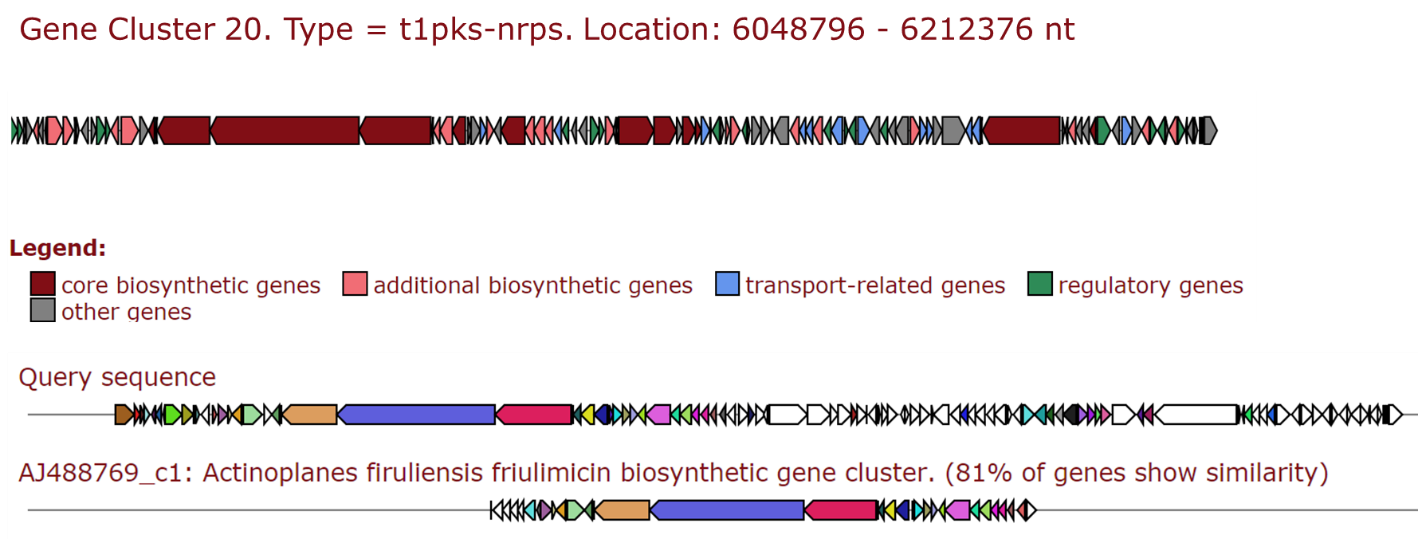


**Figure S4.**

**
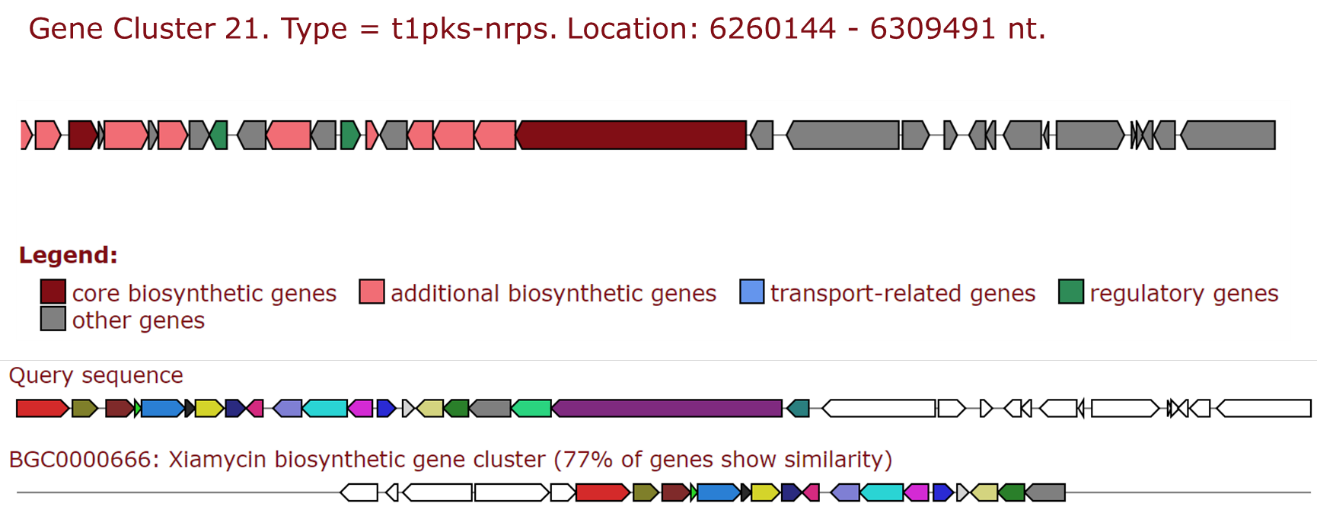
**

**Figure S5.**


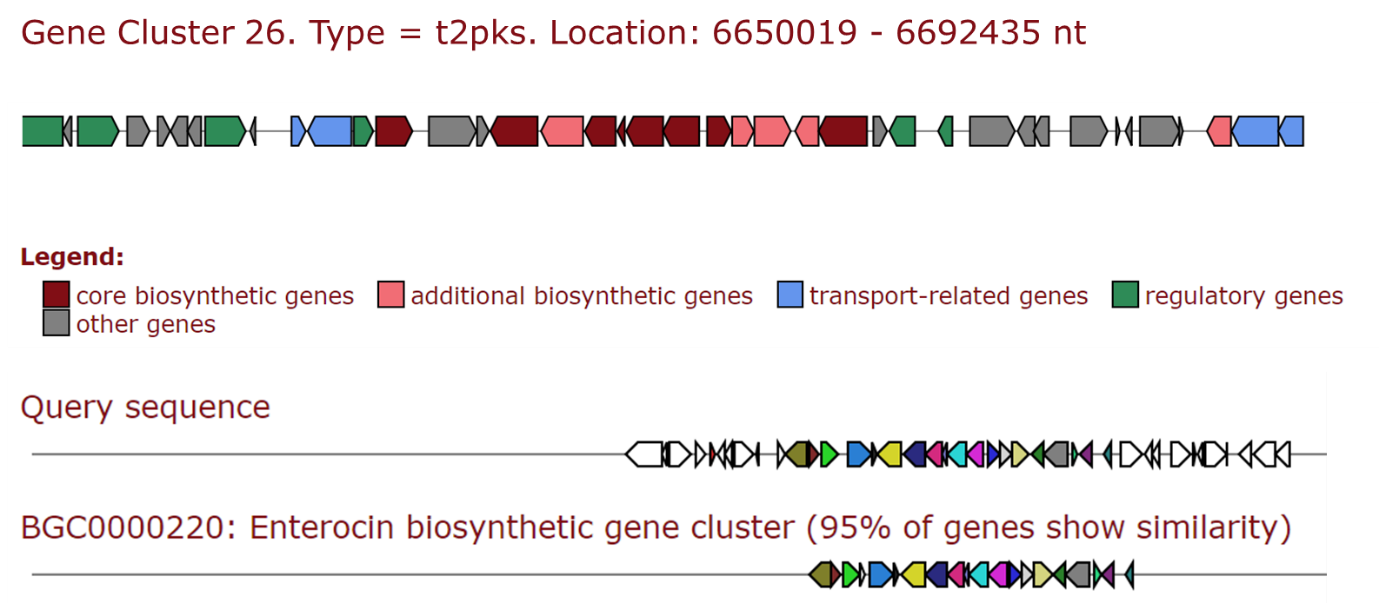


**Figure S6.**

**
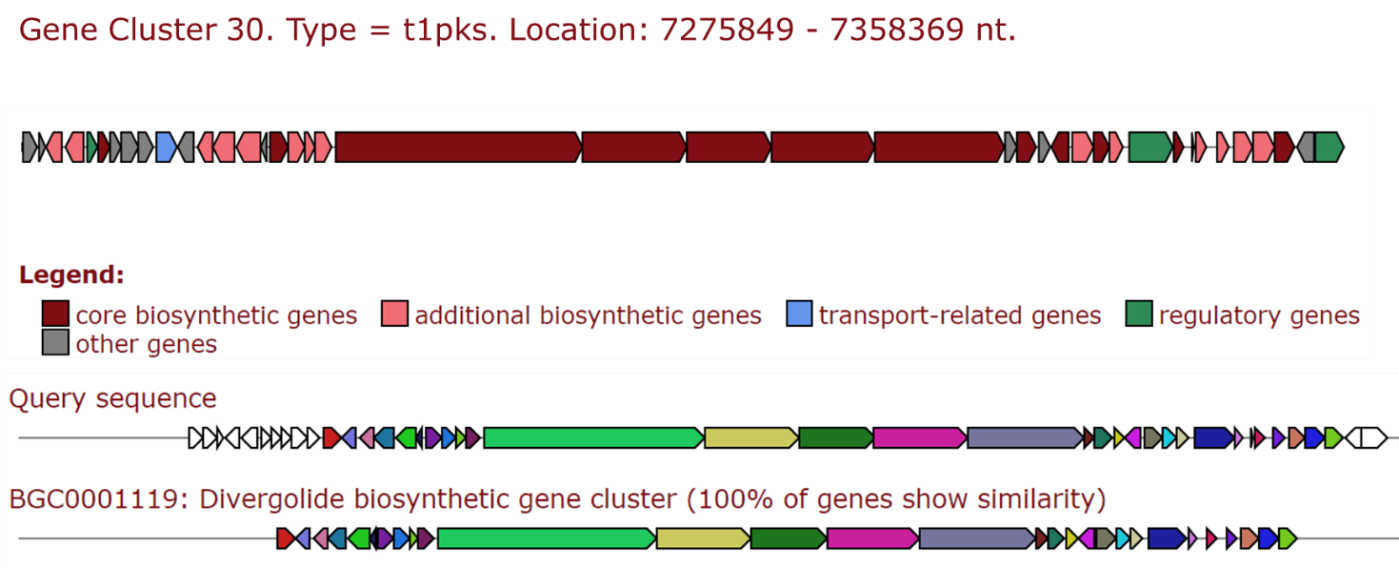
**

**Figure S7.**

**
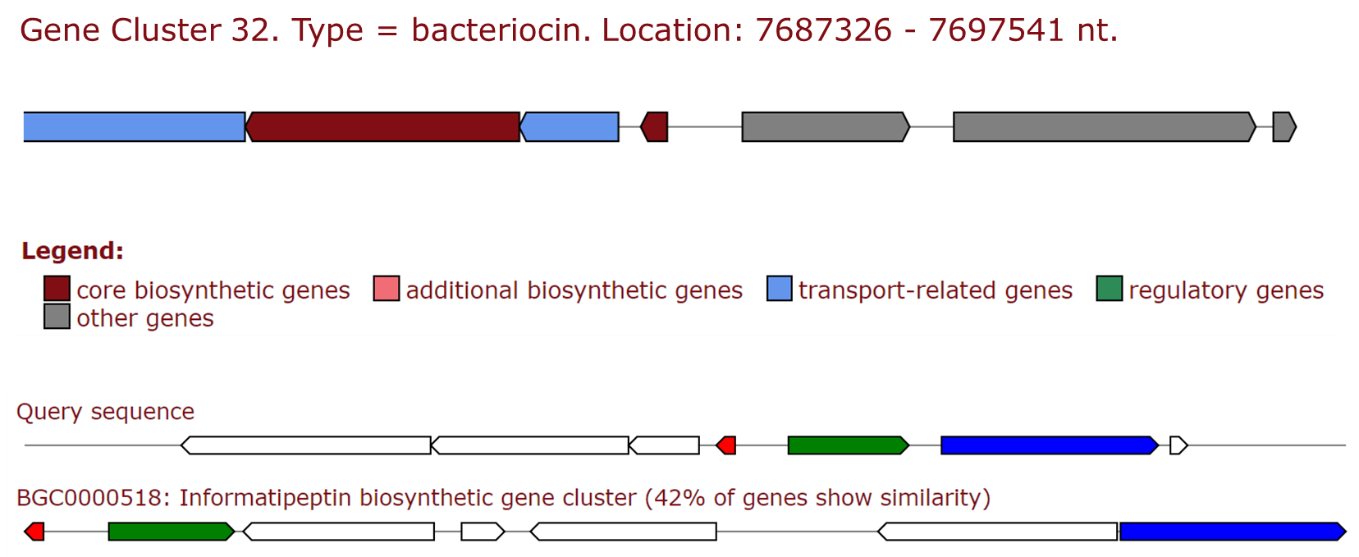
**

**Figure S8.**

**
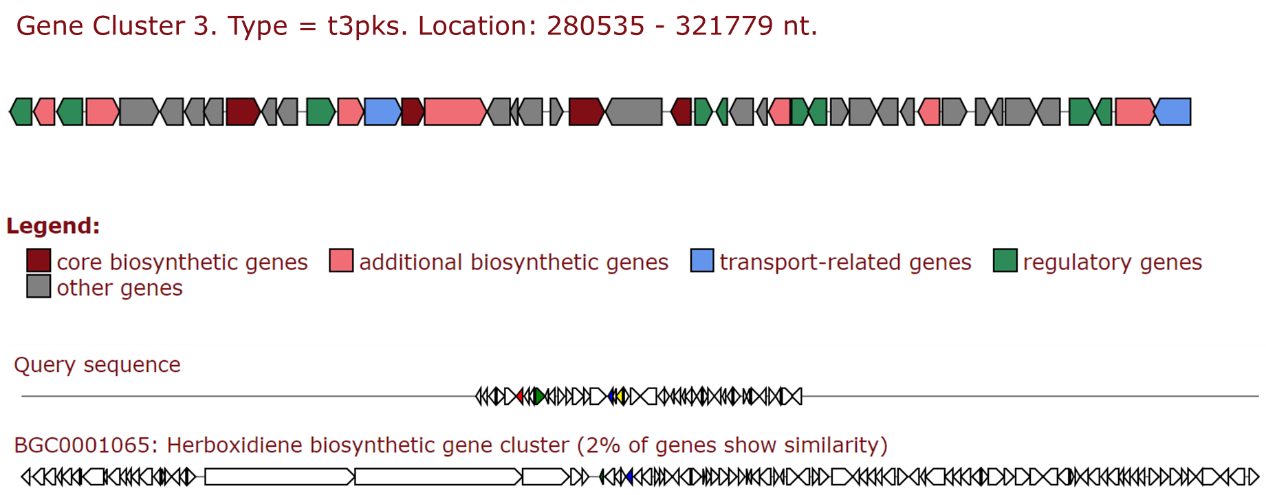
**

**Figure S9.**


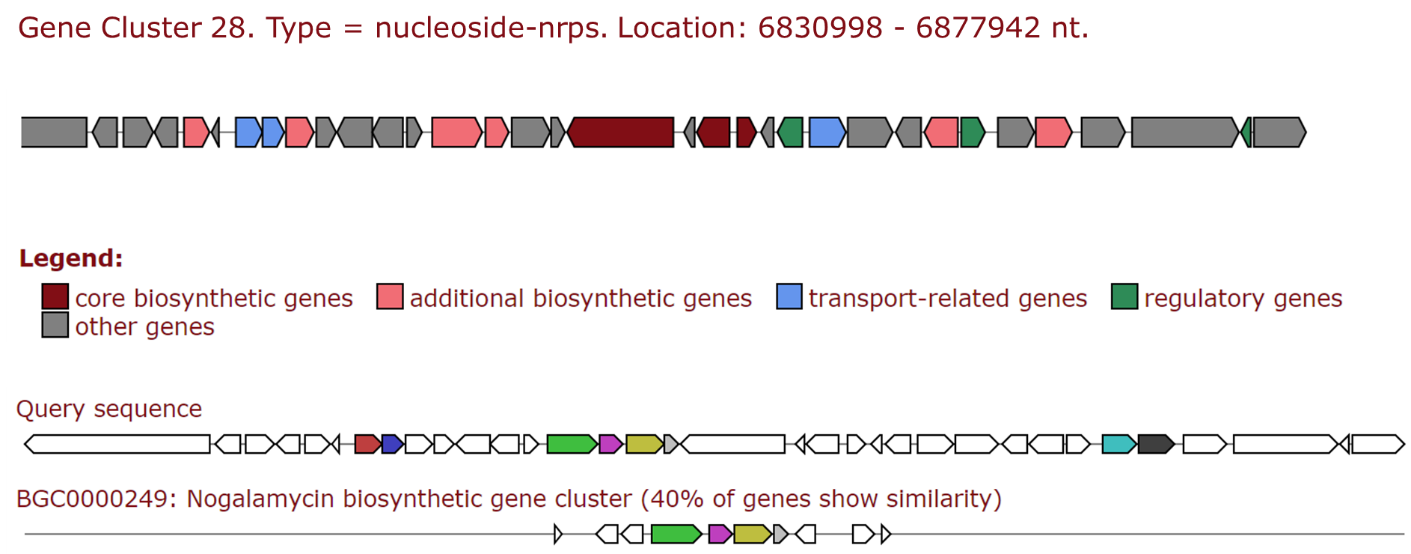


**Figure S10.**

(**A**)

**
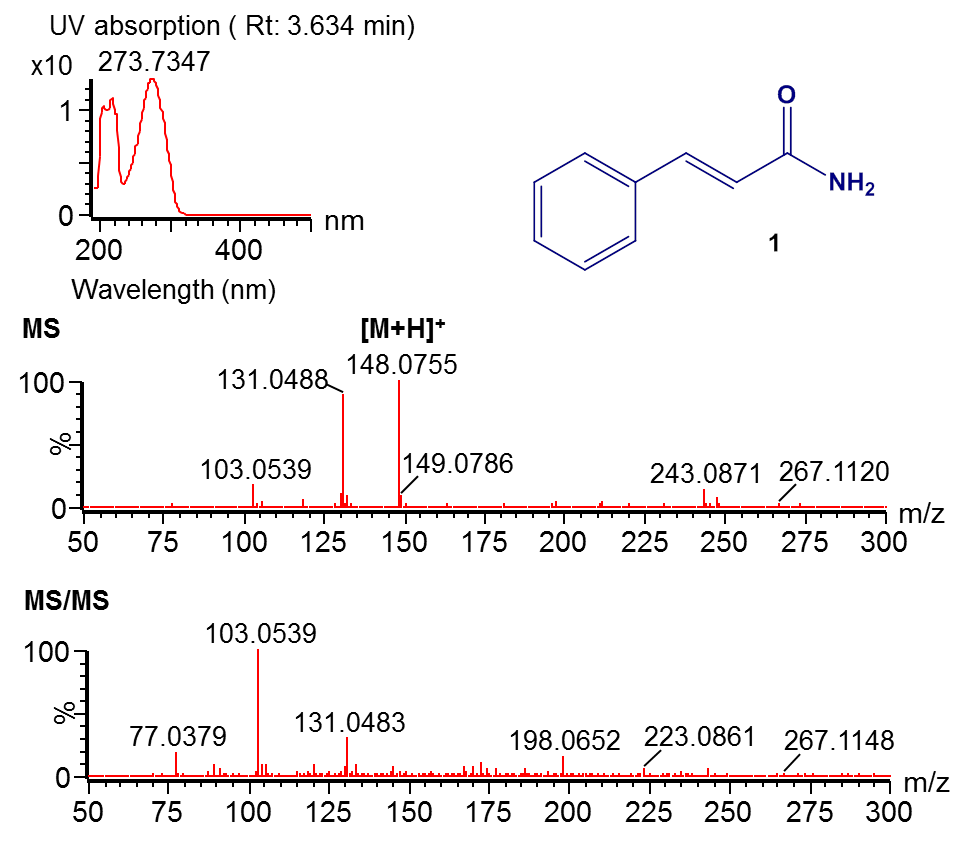
**

(**B**)

**
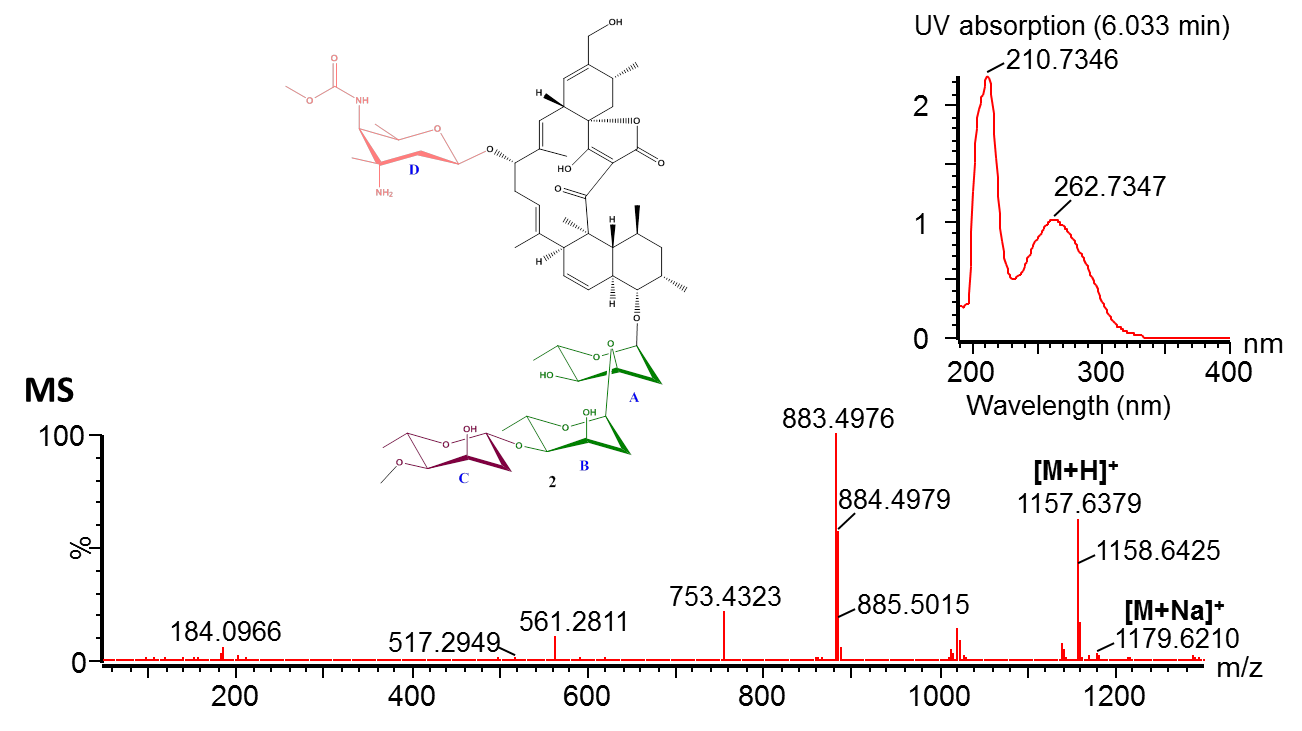
**

(**C**)

**
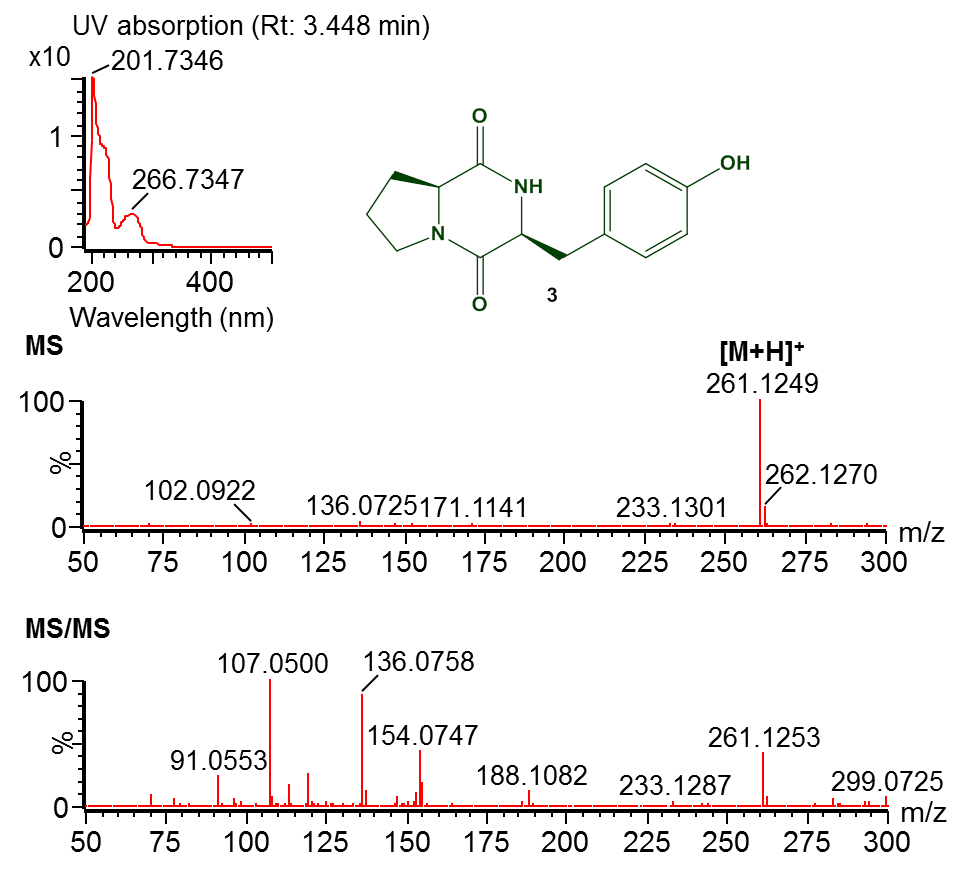
**

**Figure S11.**

**
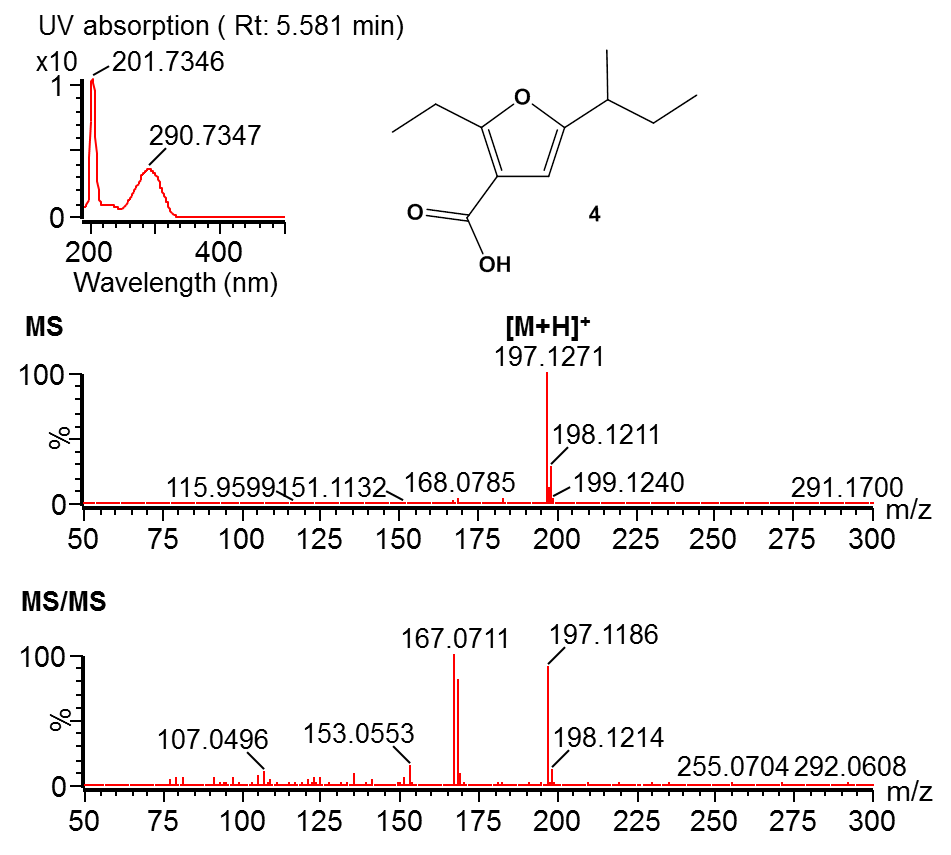
**

**Figure S12.**

**
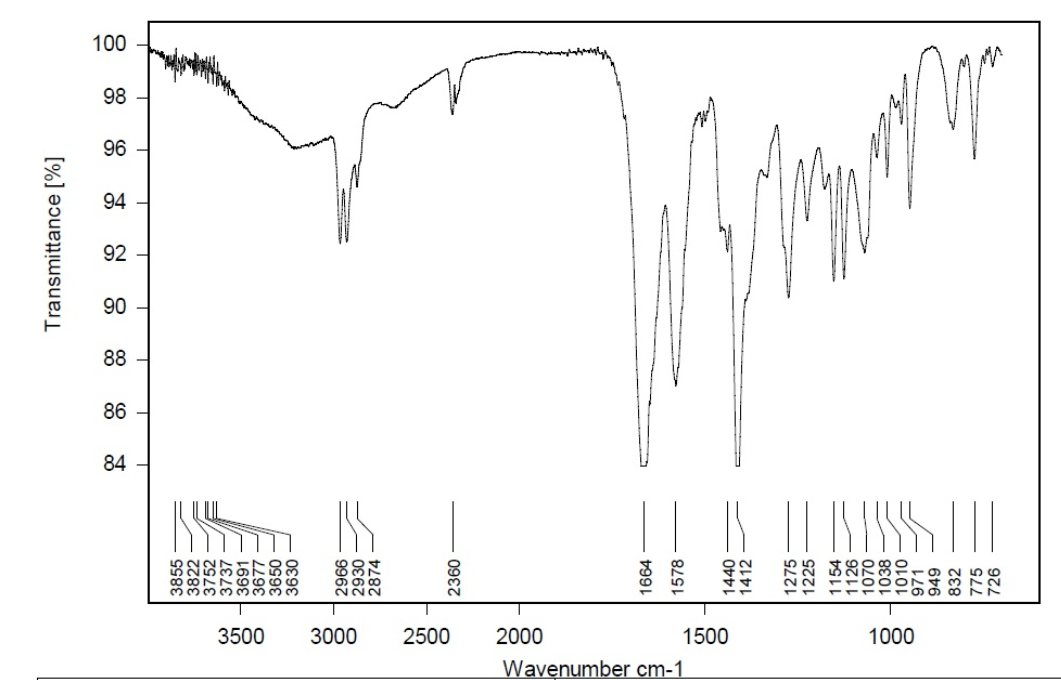
**

**Figure S13.**

(**A**)


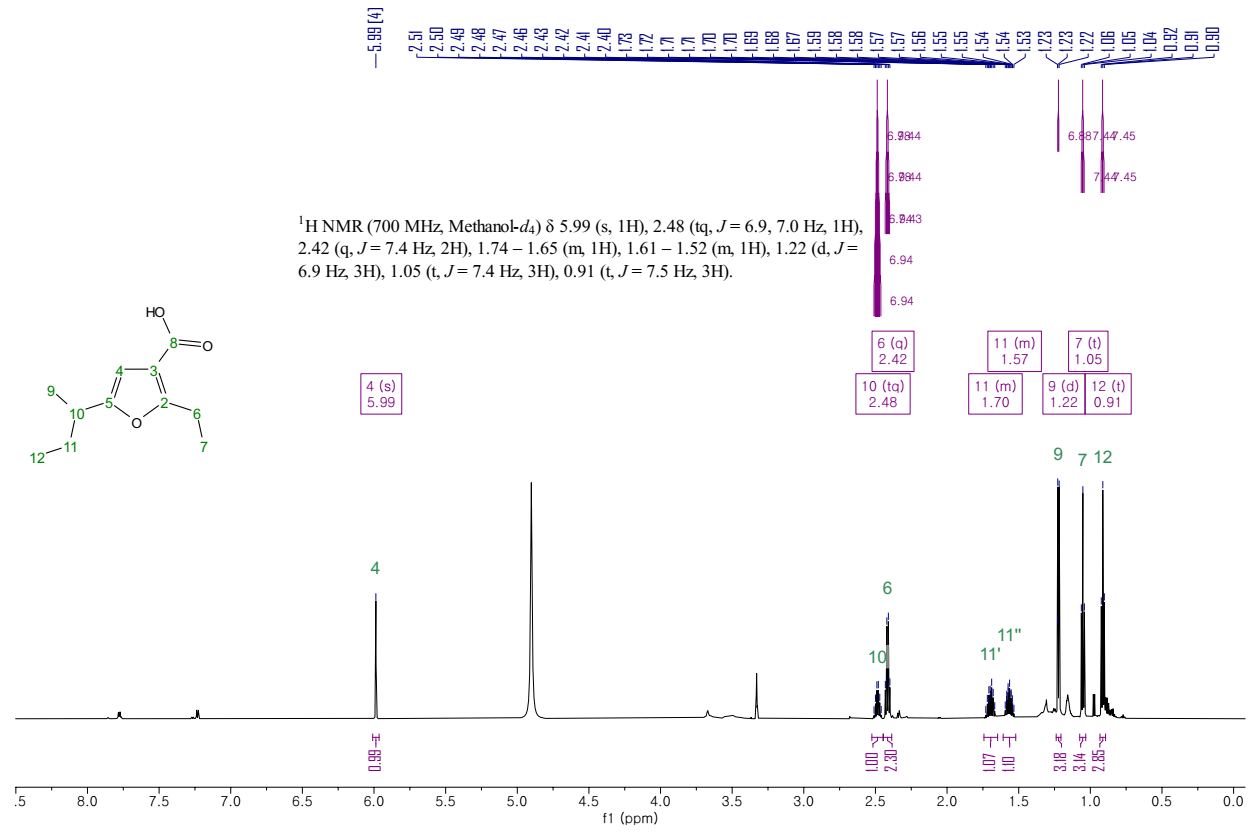


(**B**)

**
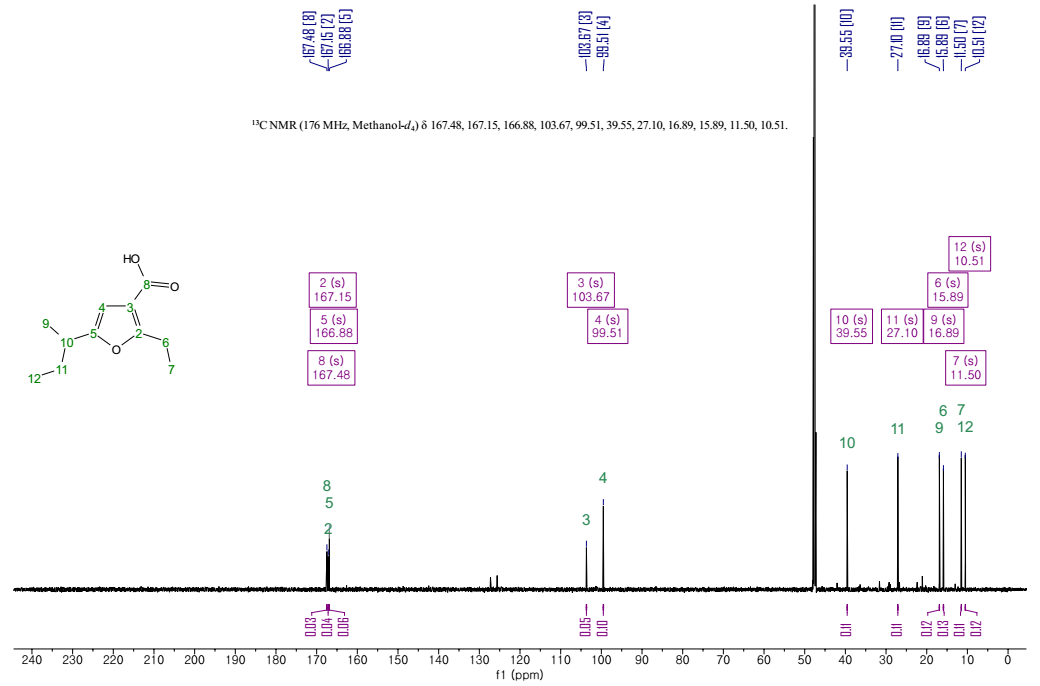
**

(**C**)

**
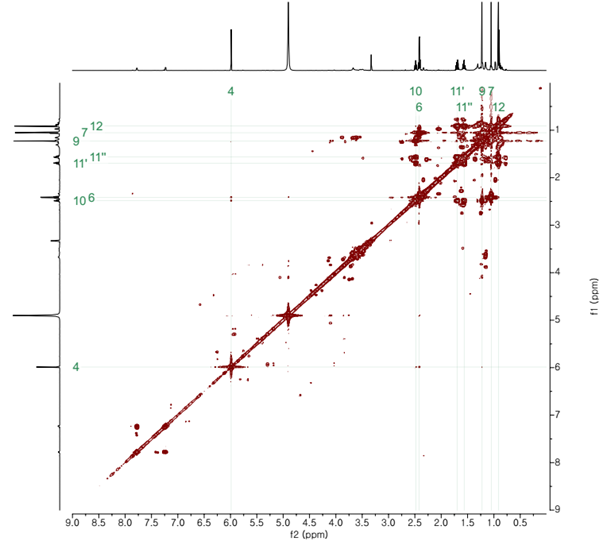
**

(**D**)

**
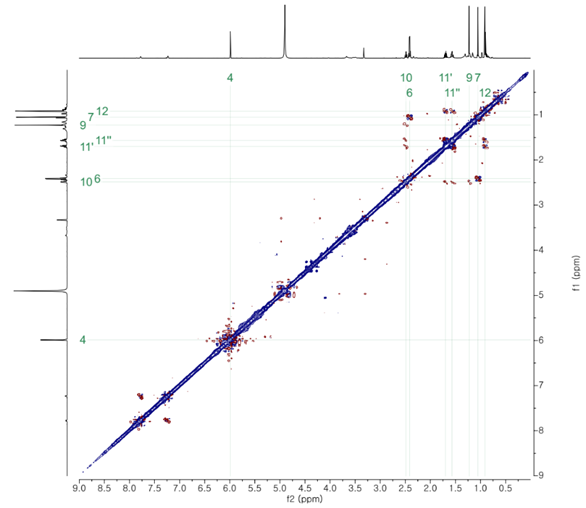
**

(**E**)

**
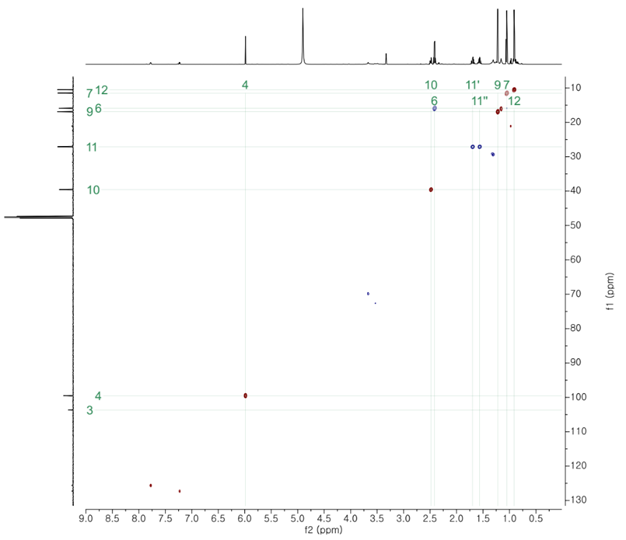
**

(**F**)

**
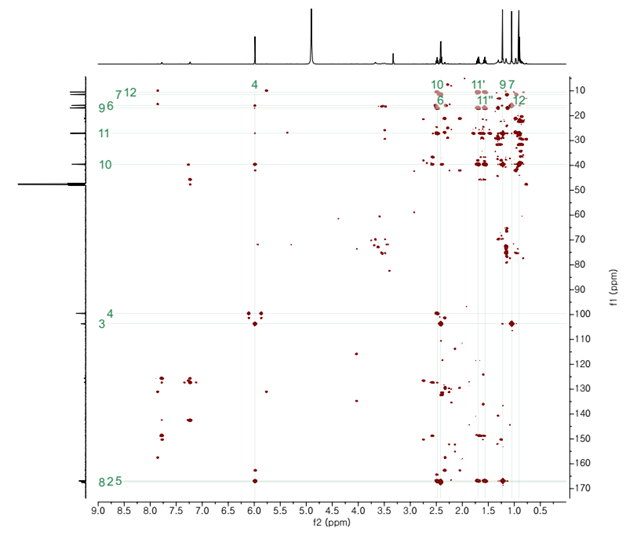
**

**Figure S14.**


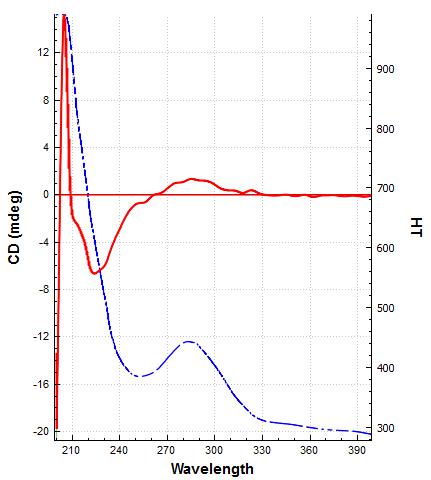


**Figure S15.**

(**A**)

(**B**)

**
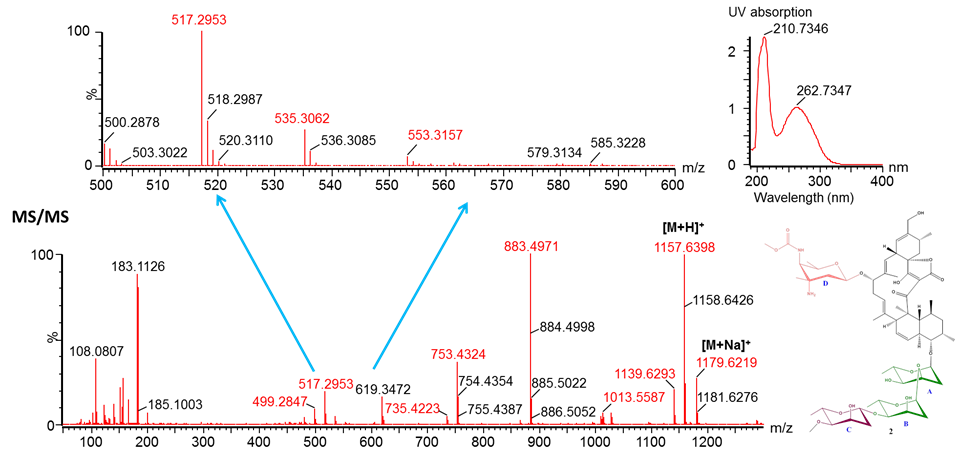
**

(**C**)

**
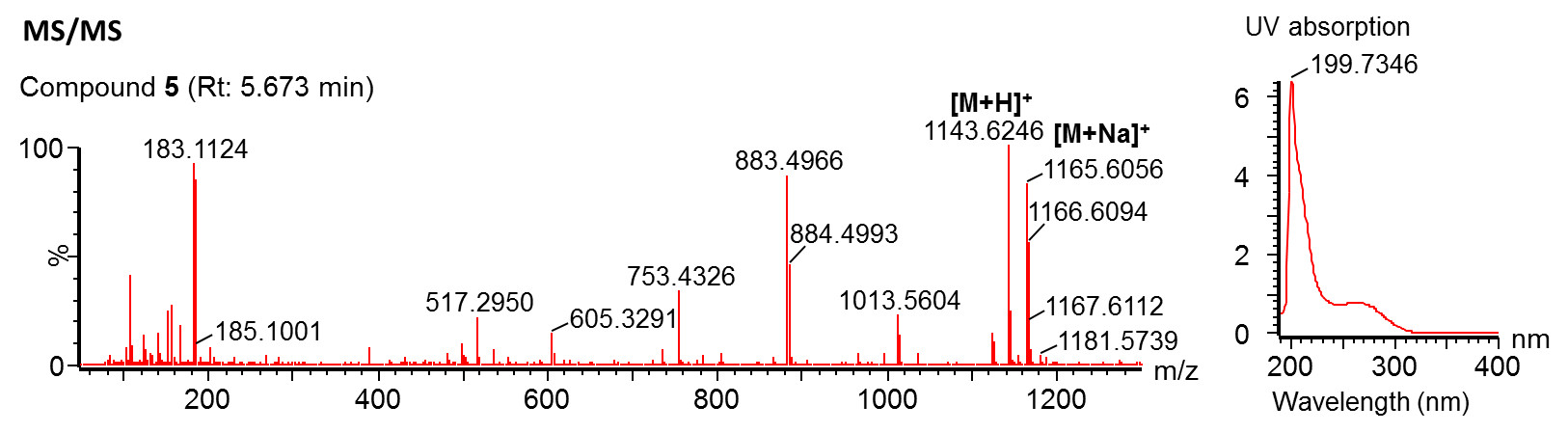
**

(**D**)

**
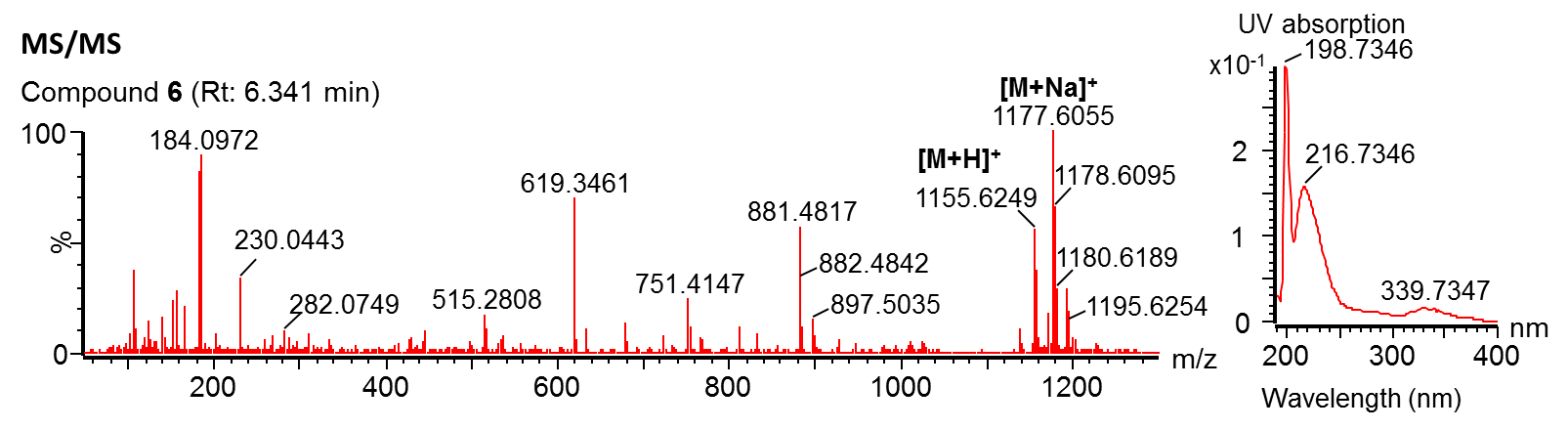
**

(**E**)

**
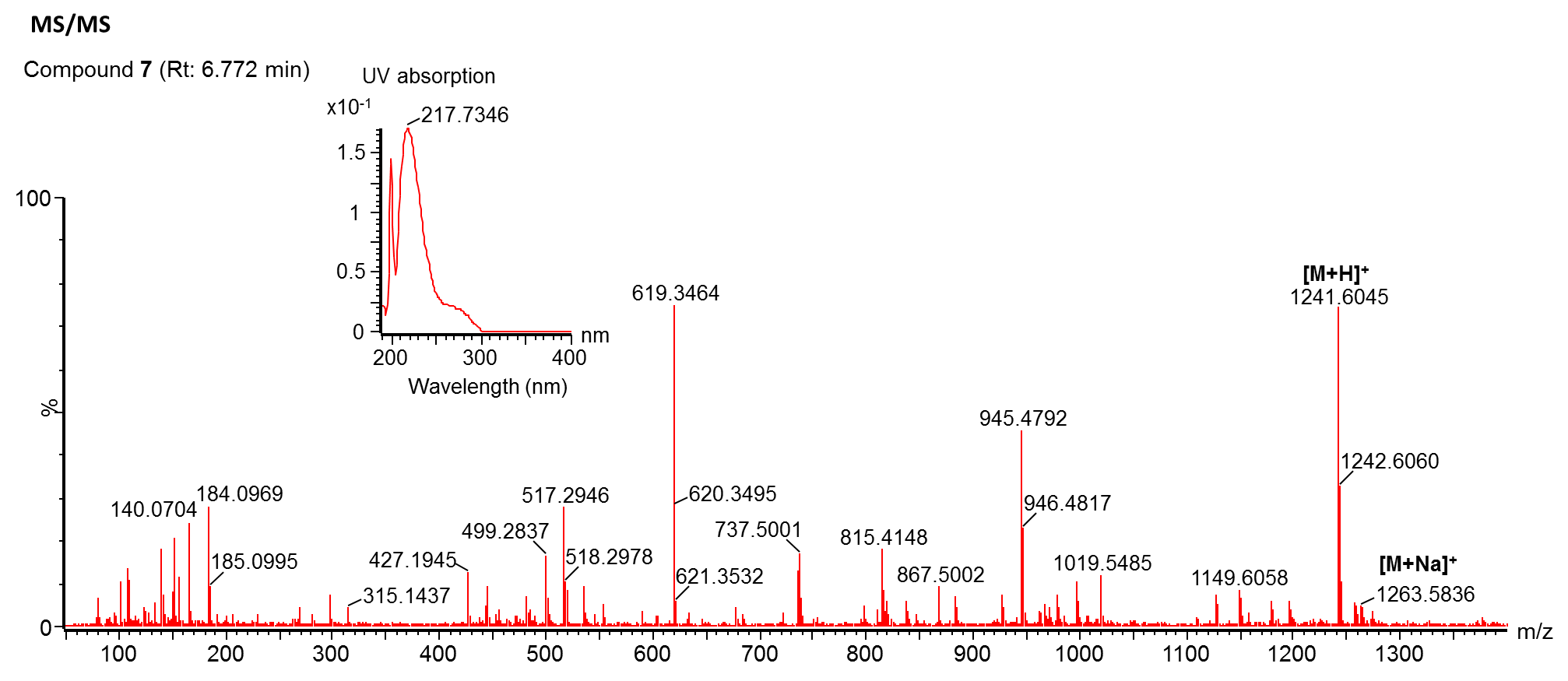
**

(**F**)

**
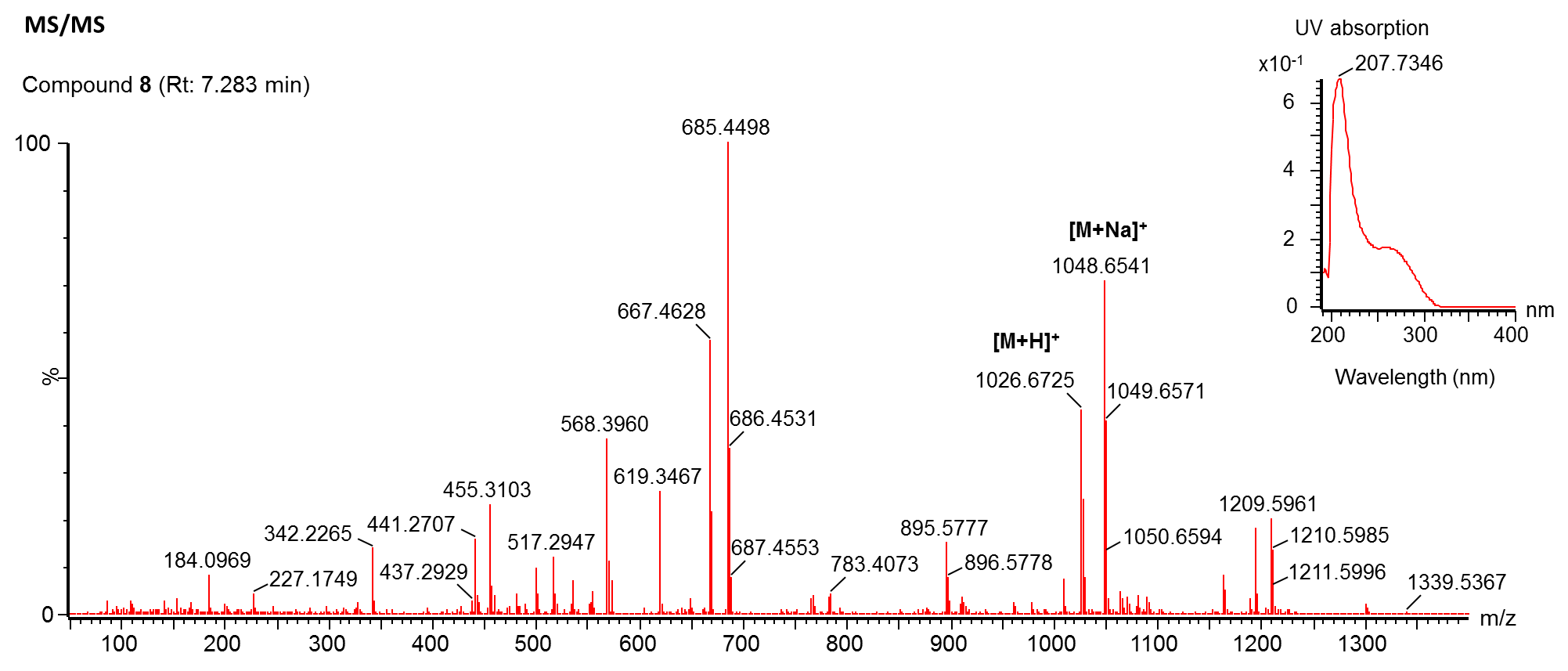
**

**Figure S16.**
